# Subdominance and poor intrinsic immunogenicity limit humoral immunity targeting influenza HA stem

**DOI:** 10.1172/JCI123366

**Published:** 2019-01-22

**Authors:** Hyon-Xhi Tan, Sinthujan Jegaskanda, Jennifer A. Juno, Robyn Esterbauer, Julius Wong, Hannah G. Kelly, Yi Liu, Danielle Tilmanis, Aeron C. Hurt, Jonathan W. Yewdell, Stephen J. Kent, Adam K. Wheatley

**Affiliations:** 1 Department of Microbiology and Immunology, University of Melbourne at The Peter Doherty Institute for Infection and Immunity, Melbourne, Victoria, Australia.; 2 WHO Collaborating Centre for Reference and Research on Influenza, The Peter Doherty Institute for Infection and Immunity, Melbourne, Victoria, Australia.; 3 Laboratory of Viral Diseases, National Institute of Allergy and Infectious Diseases (NIAID), NIH, Bethesda, Maryland, USA.; 4 Melbourne Sexual Health Centre and Department of Infectious Diseases, Alfred Hospital and Central Clinical School, Monash University, Melbourne, Victoria, Australia.; 5 Australian Research Council (ARC) Centre for Excellence in Convergent Bio-Nano Science and Technology, University of Melbourne, Parkville, Victoria, Australia.

**Keywords:** Immunology, Vaccines, Adaptive immunity, B cells, Influenza

## Abstract

Both natural influenza infection and current seasonal influenza vaccines primarily induce neutralizing antibody responses against highly diverse epitopes within the “head” of the viral hemagglutinin (HA) protein. There is increasing interest in redirecting immunity toward the more conserved HA stem or stalk as a means of broadening protective antibody responses. Here we examined HA stem–specific B cell and T follicular helper (Tfh) cell responses in the context of influenza infection and immunization in mouse and monkey models. We found that during infection, the stem domain was immunologically subdominant to the head in terms of serum antibody production and antigen-specific B and Tfh cell responses. Similarly, we found that HA stem immunogens were poorly immunogenic compared with the full-length HA with abolished sialic acid binding activity, with limiting Tfh cell elicitation a potential constraint to the induction or boosting of anti-stem immunity by vaccination. Finally, we confirm that currently licensed seasonal influenza vaccines can boost preexisting memory responses against the HA stem in humans. An increased understanding of the immune dynamics surrounding the HA stem is essential to inform the design of next-generation influenza vaccines for broad and durable protection.

## Introduction

Influenza viruses cause significant global morbidity and mortality through seasonal epidemics and periodic pandemics. The effectiveness of influenza vaccination is limited by the focusing of humoral immunity on a cluster of highly mutable epitopes in the globular head domain of the viral hemagglutinin (HA). This results in neutralization that is notoriously strain specific and leaves human populations vulnerable to antigenically novel viruses arising from antigenic drift or emerging from zoonotic reservoirs. Expanding vaccine immunity beyond classical, immunodominant variable epitopes is critical for the development of more broadly effective vaccines.

HA epitopes in the conserved HA stem that allow neutralization of highly diverse influenza strains by antibodies have been identified (1–5). While targeting the stem has energized efforts to develop universal influenza vaccines (6, 7), stem-specific antibodies in humans are generally found at low serological concentrations (8), with only limited increases after seasonal immunization or infection (3, 9, 10). Infection or immunization with highly novel influenza viruses, for example the 2009 pandemic H1N1 or avian H5N1 virus, can drive the preferential expansion of stem-specific memory B cells and serum antibodies (10–12). However, subsequent reexposure to matched HA reestablishes humoral responses dominantly targeting the variable HA head domain (10, 13). The intrinsic immunological hierarchy that exists between the stem and head regions of HA is further evident when site-directed glycosylation of the head domain resulted in an 8-fold enhancement of stem-specific antibody titers relative to responses induced by an unmodified HA counterpart (14). The immunological subdominance of stem-specific B cell responses constitutes a major obstacle in efficiently targeting the HA stem by vaccination.

Despite the extensive characterization of humoral immunity to influenza spanning many decades, the mechanisms driving the establishment and maintenance of immunodominance hierarchies of HA epitopes remain unclear. The polyspecificity (or self-reactivity) of stem-binding antibodies, particularly those derived from VH1-69 germlines, was flagged as potentially reducing the responsiveness of stem-specific B cells in humans (13). However, subdominance of stem responses is conserved in mice (15, 16) and macaques (17), which lack human-like VH1-69 alleles, suggesting the importance of other factors. Indeed, immunodominance of the HA globular head over the stem extends to lampreys, which have evolved unique analogs to vertebrate antibodies termed variable lymphocyte receptors (16). Andrews et al. recently reported that stem epitopes are poorly exposed on whole influenza virions relative to head epitopes, constraining recognition by human antibodies (13) and potentially contributing to immunological subdominance in vivo. These observations suggest that factors intrinsic to the immunogens, such as protein conformation and epitope accessibility, may underpin universal rules for B cell immunodominance hierarchies.

B cell–intrinsic factors may also modulate antibody responses to HA. The frequency of naive precursors is known to contribute to immunodominance patterns in cytotoxic CD8^+^ T cells responding to viral infection (18, 19). It remains possible that analogous differences in naive and/or memory B cell frequencies may contribute to stem versus head immunodominance. However, the dynamics of polyclonal B cell selection of complex antigens such as HA suggests that precursor frequencies or initial B cell receptor (BCR) avidities fail to explain the positive selection within germinal center (GC) responses or contribution to serum antibody levels (20). Alternatively, qualitative differences may also be critical; for instance, stem-specific B cells may be recruited less efficiently into nascent immune responses, respond less robustly to antigenic stimuli, or be impaired in their ability to proliferate and/or terminally differentiate into plasma cells, which seed the bone marrow and provide a lasting source of serum antibodies. Finally, extrinsic factors such as the availability of T follicular helper (Tfh) cell responses may also be a limiting factor in sufficiently stimulating a robust proliferation of stem-specific B cells in active GCs. Overall, a mix of immunogen-intrinsic (concentration, localization, conformation of HA), B cell–intrinsic (frequency, phenotype, proliferative capacity, trafficking, polyspecificity), and extrinsic factors (CD4^+^ T cell help) probably combine to underpin the predominance of head versus stem humoral responses to influenza HA.

Here we demonstrate in naive mouse and monkey models that immunological subdominance of the HA stem is established early during primary infection. In contrast to head-specific responses, stem-specific B cells fail to expand, be recruited to secondary lymphoid tissues, or seed the plasma cell compartment in the bone marrow following infection, despite high concentrations of HA stem antigen at the site of infection. Further, we show that HA stem immunogens elicited poor stem-specific responses in naive or preimmune animals but that responses could be restored when physically linked to either the head domain or a keyhole limpet hemocyanin (KLH) carrier protein. Finally, we confirmed that currently licensed seasonal influenza vaccines can drive reexpansion of stem-specific memory B cells and elevated stem-specific serum antibody levels in humans. A greater mechanistic understanding of the drivers of immunogenicity and immunodominance will inform vaccine strategies targeting HA stem epitopes as a pathway to universal influenza protection.

## Results

### Stem-specific B cell responses are highly subdominant during primary infection.

**Figure 1:**
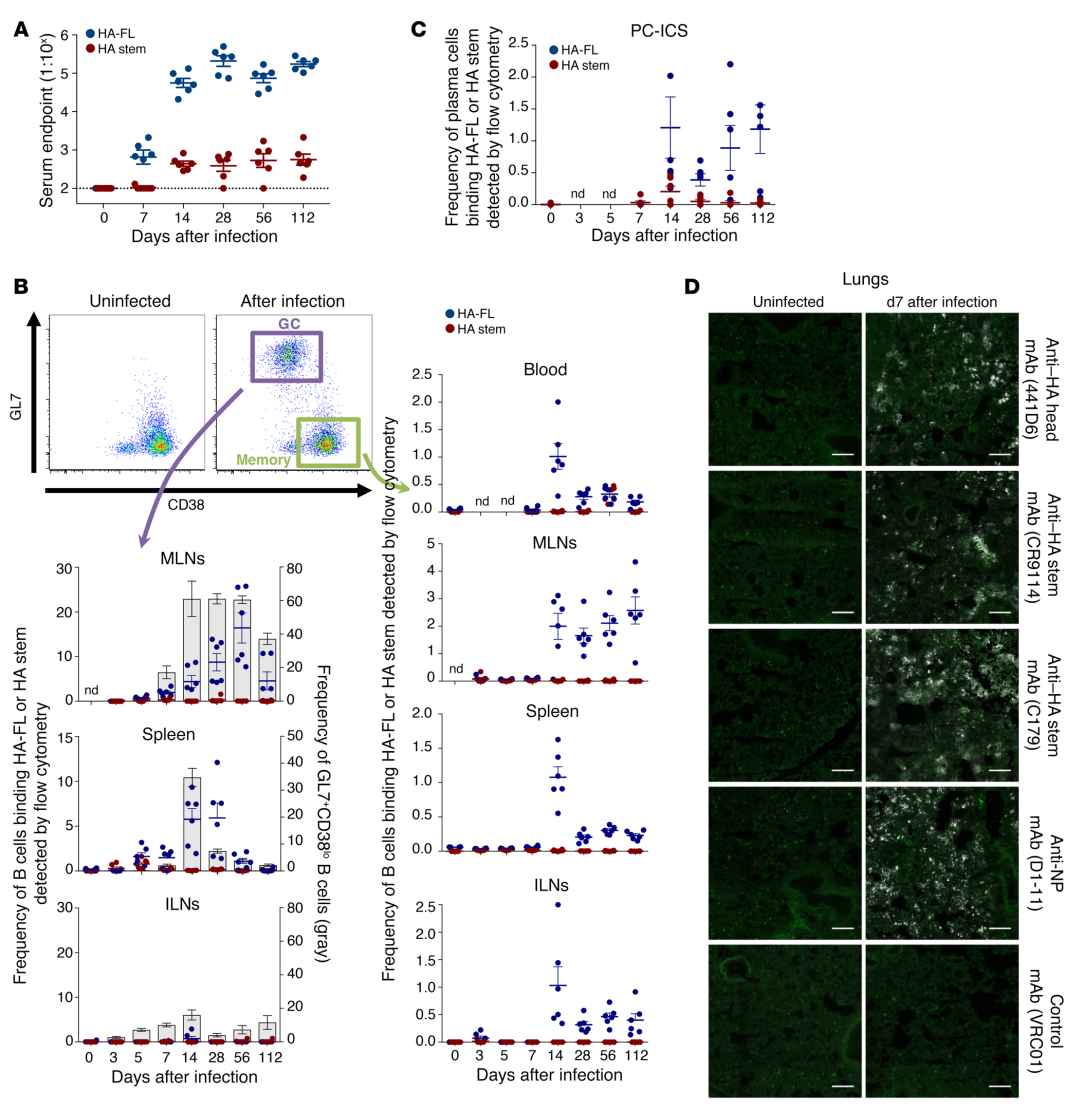
Serological and B cell responses in experimentally infected mice. (**A**) Serum endpoint total IgG titers were measured by ELISA using HA-FL (blue) or stabilized HA stem (red) in mice infected intranasally with PR8 (*n* = 6 per time point). Dotted lines denote the detection cutoff (1:100 dilution). Data represent the mean ± SEM. (**B**) Frequency of GC B cells (B220^+^IgD^–^CD38^lo^GL7^+^) and memory B cells (B220^+^IgD^–^CD38^hi^GL7^–^) binding HA-FL (blue) or HA stem (red) (*n* = 6). Data represent the mean ± SEM. (**C**) Frequency of plasma cells (CD138^+^B220^–^IgD^–^) binding HA-FL (blue) or HA stem (red) (*n* = 6). Data represent the mean ± SEM. (**D**) HA bioavailability visualized by monoclonal anti–HA head or anti–HA stem antibody staining (white) and B220^+^ B cell staining (green). Scale bars: 100 μm.

We first examined the spatial and temporal dynamics of HA and stem-specific humoral immunity during primary H1N1 influenza infection in C57BL/6 mice intranasally infected with A/Puerto Rico/08/1934 (PR8). To enable the study of HA-specific immunity, we first generated full-length HA (HA-FL) and stabilized HA stem proteins as described previously (6, 21), and then confirmed antigenic specificity by binding to well-characterized monoclonal antibodies (Supplemental Figure 1; supplemental material available online with this article; https://doi.org/10.1172/JCI123366DS1). After a single, nonlethal infection, we observed the rapid development of a HA-FL–specific serum antibody response by day 7 after infection that peaked by day 28 and was maintained at high titers out to day 112 (Figure 1A). In contrast, serum antibodies specific for the HA stem were first detectable only by day 14 and were then maintained at serum titers that were over 100-fold lower than those for antibodies against HA-FL.

For the direct assessment of influenza HA-FL– and HA stem–specific immunity at the B cell level, we examined the frequency and specificity of memory and GC B cells using PR8 HA-FL or PR8 HA stem flow cytometric probes (gating, Supplemental Figure 2). Within the mediastinal lymph node (MLN), which drains the lungs and where influenza-specific B cell responses are initiated following infection (22–24), lymphoid remodeling and GC responses were rapidly established (Supplemental Figure 3). The HA-FL and HA stem probes allowed us to simultaneously track both total GC B cell responses (B220^+^IgD^–^CD38^lo^GL7^+^) and the proportions that were HA-FL or HA stem specific. We found that both total GC B cells and the sizable subpopulation of cells that were HA-FL specific expanded out to day 14 and were maintained at elevated levels until day 112 (Figure 1B; see representative plots in Supplemental Figure 4). During this time, B cell selection and antibody affinity maturation to HA likely continues within the MLNs (23). Within the spleen, major remodeling and significant GC expansion occurred after infection (Supplemental Figure 5), with the frequency of HA-specific B cells within the GC population reaching approximately 5% by day 14, before waning over time. Neither GC formation nor expansion of HA-specific GC B cells was observed within the nondraining inguinal lymph nodes (ILNs).

HA-specific memory B cells (B220^+^IgD^–^CD38^hi^GL7^–^) peaked in the blood on day 14, before rapidly contracting to a stable level of approximately 0.3% of the total blood memory B cell population that was maintained out to day 112. We observed similar dynamics and resting frequencies within the memory cell populations in the spleen and nondraining ILNs. In contrast, HA-specific MLN memory B cells were rapidly expanded by day 14 but were maintained at high frequencies (~2%) out to day 112. Consistent with previous observations that the frequency of lymph node B cells predicts serum antibody immunodominance (15), our observations of low serum antibodies specific for HA stem coincided with very limited numbers of HA stem–specific B cells detected within the blood or lymphoid tissues by flow cytometry. Likewise, while HA-specific B cells could be readily visualized by confocal microscopy within the MLNs (Supplemental Figure 6) or spleen (Supplemental Figure 7) of infected mice, we detected little to no staining for B cells binding the HA stem either localized in the GC or distributed within the tissues.

To enumerate antigen-specific antibody-secreting cells (ASCs) or plasma cells within the bone marrow of infected mice, we devised an intracellular staining protocol of CD138^+^ plasma cells with the HA-FL and HA stem probes (Figure 1C; see gating in Supplemental Figure 8). In line with the low titers of stem antibodies, few stem-specific plasma cells were evident, while plasma cells secreting antibodies specific for HA-FL were readily detected. The narrow epitope specificity of the PR8 HA–specific antibody and B cell response was further confirmed using a HA probe derived from SV12 virus (15, 25), which carries 12 amino acid substitutions enabling near-total escape from serological recognition at canonical epitopes (Supplemental Figure 9). Thus, in line with findings from previous studies (15), we found that primary PR8 infection in mice was dominated serologically and at the cellular level by B cells that target canonical epitopes surrounding the receptor binding site, despite the presence of bioavailable stem epitopes within the lungs of the infected mice (Figure 1D). Subdominance of the pandemic H1N1 (pdmH1N1) HA stem was additionally confirmed using mice infected with A/California/04/2009 (CA09) (Supplemental Figure 10). To confirm that these observations were not exclusive to the C57BL/6 mouse strain, we examined infected BALB/c mice and found a similar predominance of HA-FL over HA stem responses on day 14 after infection (Supplemental Figure 11, A and C).

**Figure 2:**
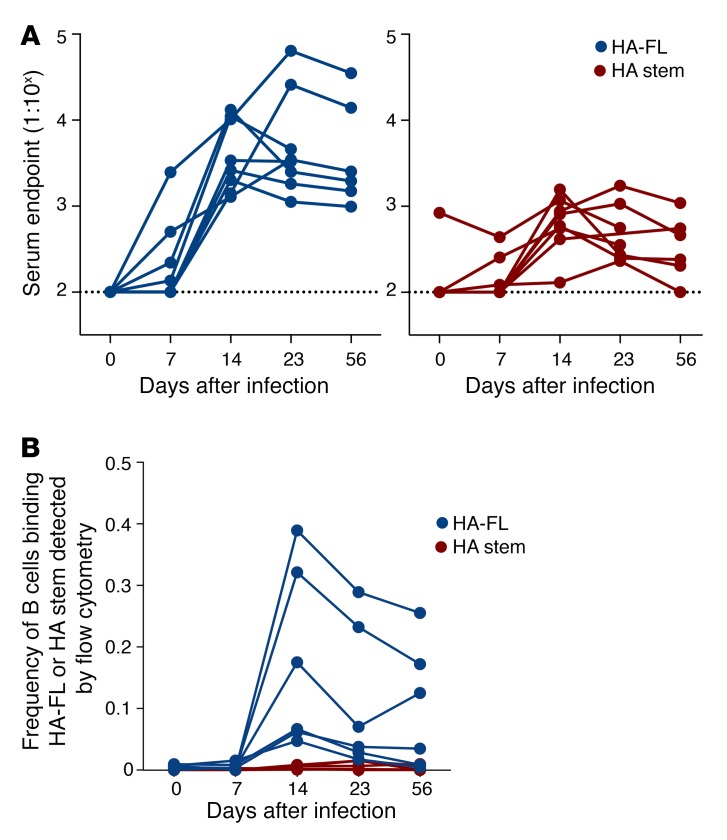
Serological and B cell responses in experimentally infected macaques. (**A**) Serum endpoint total IgG titers were measured by ELISA using CA09 HA-FL (blue) or stabilized CA09 HA stem (red) in macaques (*n* = 8) infected intranasally with A/Auckland/1/2009. Note that 2 animals were sacrificed on day 23. Dotted lines denote the detection cutoff (dilution 1:100). (**B**) Frequency of IgG^+^ memory B cells (CD19^+^IgD^–^IgG^+^) binding CA09 HA-FL (blue) or stabilized CA09 HA stem (red) was measured by flow cytometry within cryopreserved PBMC samples from infected macaques (*n* = 6). Note that the 2 animals sacrificed on day 23 were excluded.

To extend these findings to a more relevant animal model for human influenza, we infected 8 pigtail macaques (*Macaca nemestrina*) with pdmH1N1 A/Auckland/1/2009, which is antigenically indistinguishable from CA09. The mean serum endpoint levels at the peak of the response (day 14 after infection) were approximately 1:3800 and approximately 1:600 for HA-FL and HA stem responses, respectively (Figure 2A). While the mean HA-FL responses were maintained out to day 56, HA stem responses decreased by 2-fold (~1:300) relative to the titers on day 14. HA stem responses were also 12-fold lower than HA-FL responses on day 56.

We examined the frequency of memory B cells (CD19^+^IgD^–^IgG^+^) in cryopreserved PBMC samples of infected macaques using CA09 HA-FL or HA stem flow cytometric probes (Figure 2B). B cells specific for HA-FL were detectable on day 14 (~0.1%), coinciding with the appearance of serological HA-FL–specific antibodies, and slightly waned by day 56. In contrast, little to no HA stem–specific B cells were detectable throughout the course of the pdmH1N1 infection. Both murine and macaque infections displayed analogous kinetics in serology and blood-circulating memory B cells specific for HA-FL, both of which peaked on day 14, indicating that the infection models were similar.

Taken together, our data across mouse and macaque models showed that the HA stem is markedly immunologically subdominant at both cellular and serological levels during primary influenza infection in naive animals.

### The HA stem displays limited immunogenicity in isolation.

**Figure 3:**
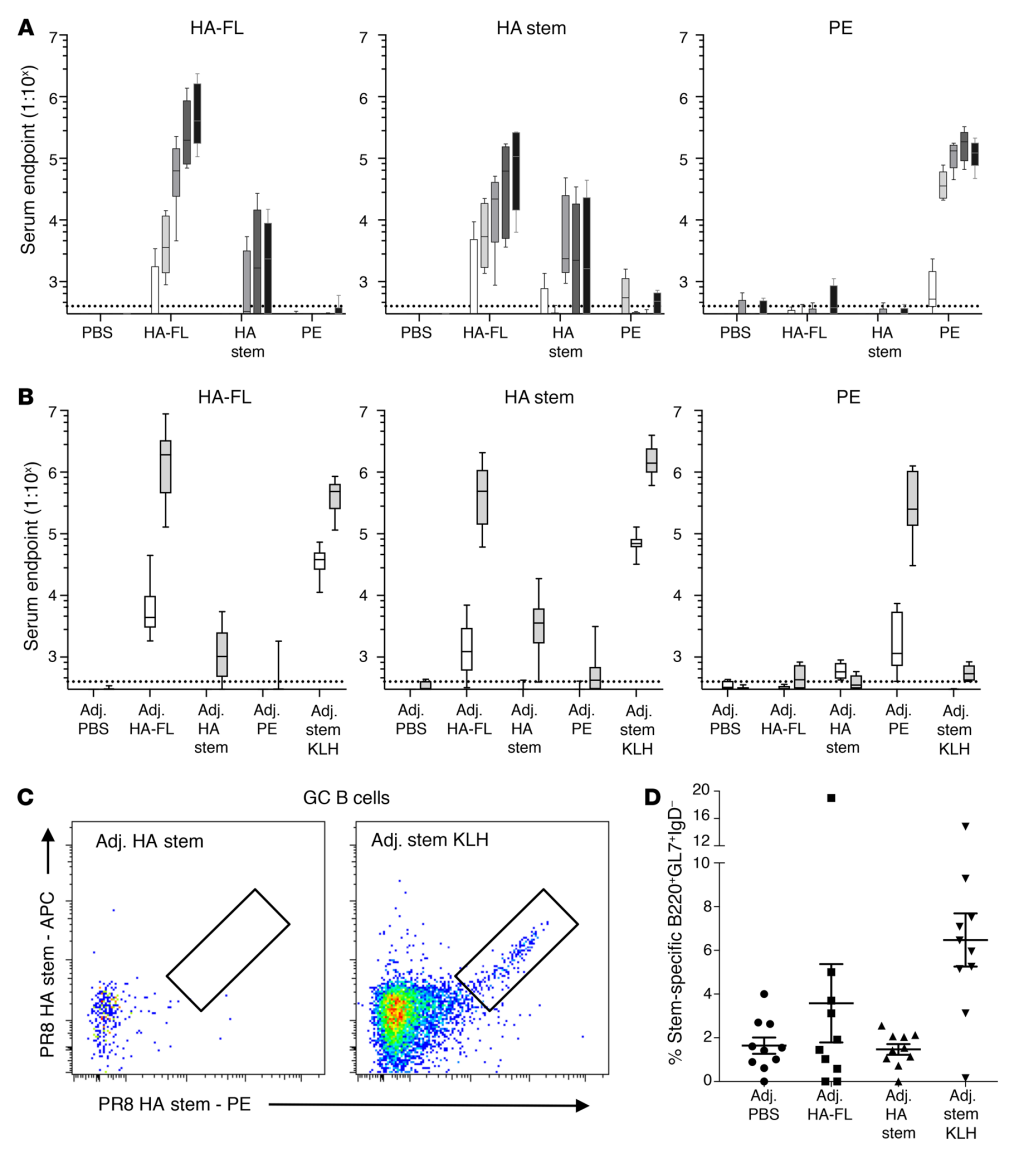
Serological and B cell responses in primary vaccinated mice. Serum endpoint total IgG titers were measured by ELISA using HA-FL, HA stem, or PE proteins in mice that were immunized (**A**) 5 times with unadjuvanted immunogens or (**B**) 2 times with adjuvanted (Addavax) immunogens (*n* = 10 from 2 independent experiments with groups of 5 animals). Mice were immunized at 3-week intervals, and serum was collected every 2 weeks after immunization. Dotted lines denote the detection cutoff (dilution 1:400). Box boundaries represent 25^th^ and 75^th^ percentiles, the inner line represents the median, and whiskers represent minimum and maximum values. (**C**) Representative flow cytometric plots and (**D**) frequency of GC B cells (B220^+^IgD^–^CD38^lo^GL7^+^) from mice vaccinated once with adjuvanted (Addavax) immunogens double stained with HA stem probes (PR8) (*n* = 10 from 2 independent experiments with groups of 5 animals). Data indicate the mean ± SEM. Adj., adjuvant.

We next examined HA-specific humoral responses in the context of immunization. C57BL/6 mice were repeatedly immunized intramuscularly in the absence of adjuvant with HA-FL, HA stem, or controls (PBS, phycoerythrin [PE]). We detected a steady increase in PE-specific serum antibody in PE-immunized mice (Figure 3A), with boosting evident for 3 immunizations until a plateau was reached at a serum dilution of approximately 1:10^5^. Likewise, vaccination with HA-FL drove a steady increase in HA-FL serum titers for all 5 immunizations, peaking at approximately 1:10^6^ after the final boost. Sequential HA-FL immunization elicited serum antibodies against the HA stem at levels that were approximately 1 log lower (peaking at ~1:10^5^) than HA-FL serum titers. This observation indicates that HA-FL immunization partially overcomes stem subdominance. Interestingly, repeated vaccination with HA stem was comparatively poorly immunogenic, with undetectable stem serum antibodies until the third immunization, peaking at a titer of approximately 1:10^4^.

Limited stem immunogenicity was maintained even when immunogens were formulated with the adjuvant Addavax, an MF59 analog. Two immunizations were sufficient to elicit strong serum antibody responses (~1:10^6^) for HA-FL or PE (Figure 3B). Analogous to the unadjuvanted regimen, stem-specific serum antibodies were detectable at high levels of approximately 1:10^6^ when immunizations were performed with HA-FL with Addavax. In contrast, only modest titers (~1:10^4^) of stem serum antibodies could be elicited by 2 injections of HA stem protein, despite inclusion of the adjuvant. We repeated the HA stem vaccinations with 4 additional adjuvants and via subcutaneous immunization but found that these regimens overall failed to rescue the HA stem serum antibody response (Supplemental Figure 12). We found that the poor immunogenicity of stem was relieved by covalently coupling it to KLH. Immunization with the conjugate in Addavax generated a very high titer of a stem-specific serum antibody response (Figure 3B), along with a high KLH-specific serum antibody response (Supplemental Figure 11B). While BALB/c mice vaccinated with HA-FL in Addavax showed a similar immunological dominance of HA-FL serum antibody responses, vaccination with the HA stem immunogen in Addavax elicited a stem-specific serum antibody response greater than that previously seen in C57BL/6 mice (Supplemental Figure 11, B and C).

To further dissect limited HA stem immunogenicity, we examined the recruitment of stem-specific B cells into the GCs of draining ILNs after a single immunization with Addavax-adjuvanted immunogens (Figure 3, C and D). Mirroring the serum antibody response, negligible GC recruitment of stem-specific B cells was observed with the HA stem protein alone, comparable to that seen with the PBS control. In contrast, immunization with the HA-FL protein or HA stem with a KLH carrier induced strong GC recruitment of stem-specific B cells. We further confirmed that linkage to a nanoparticle scaffold (6) relieved the poor immunogenicity of the HA stem at both the serological and B cell levels (Supplemental Figure 13). Taken together, these data suggest that the HA stem domain in isolation is intrinsically poorly immunogenic. However, strong stem-specific serum antibodies and their corresponding GC B cells can be induced when the stem protein is presented in the form of a full-length HA, when linked to a KLH carrier protein, or when displayed on the surface of a nanoparticle.

### The HA stem elicits limited Tfh responses following vaccination or infection.

Typical of protein antigens, CD4^+^ T cells are required for robust and durable serum antibody responses to HA (15, 26, 27). We therefore wondered whether the limited immunogenicity of HA stem immunogens is due to limiting Tfh responses. We stimulated draining ILN T cells from immunized C57BL/6 mice with overlapping 17-mer synthetic peptide sets encompassing the HA head (residues HA1 42-313, H3 numbering) or HA stem (HA1 0-42, 314-329, HA2 1-174) domains. Antigen-specific Tfh cells (CD3^+^CD4^+^CXCR5^++^PD1^++^) (Supplemental Figure 14A) were detected on the basis of upregulation of CD154 (CD40L) (Supplemental Figure 14B), a classical marker of CD4^+^ T cell help, or the activation-induced markers OX40, CD25, and/or ICOS, which preferentially identify antigen-specific Tfh cells compared with traditional intracellular cytokine staining (28–30) (Supplemental Figure 14, C and D).

**Figure 4:**
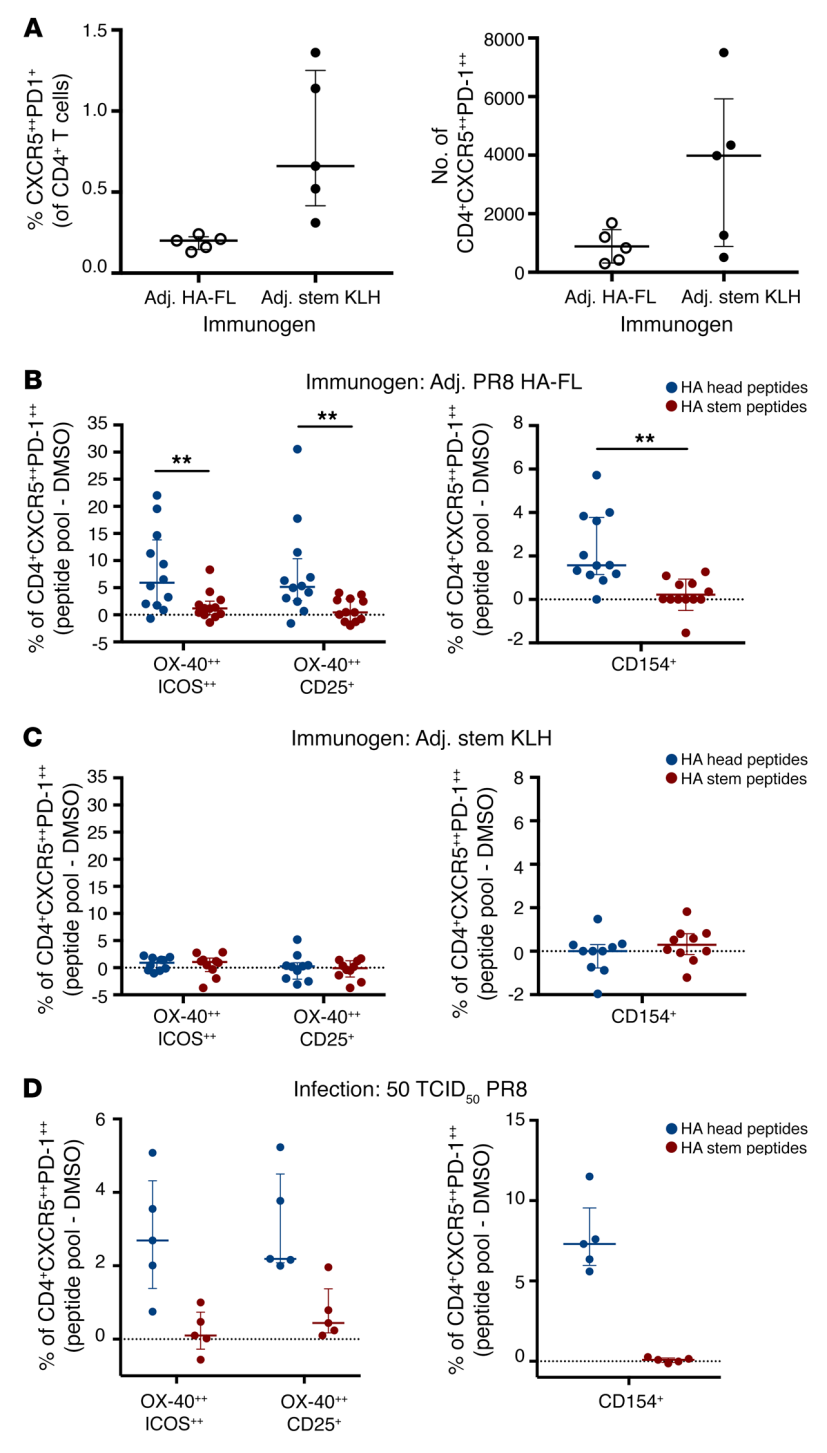
Antigen specificity of Tfh cells following immunization or infection in mice. (**A**) Tfh cells were quantified in the 2 draining ILNs on day 14 after vaccination with PR8 HA-FL or stem KLH antigens (*n* = 5). (**B**–**D**) Antigen-specific Tfh cells were identified either by OX-40 upregulation in combination with ICOS^++^ or CD25 coexpression or CD154 expression following 18 hours of stimulation with HA head or HA stem peptide pools. Antigen-specific responses are presented after background subtraction using a DMSO control (dotted line indicates no change above background). Samples were collected on day 14 after immunization or infection with PR8 HA-FL (*n* = 12), stem KLH protein (*n* = 10), or 50 TCID_50_ PR8 virus (*n* = 5). Error bars indicate the median and IQR. **P < 0.01, by Wilcoxon matched-pairs test.

Ex vivo enumeration of ILN Tfh cell populations on day 14 after immunization confirmed that sizable Tfh cell populations could be recovered from both PR8 HA-FL– and stem KLH–immunized mice (Figure 4A). HA-FL immunization induced significantly higher levels of head-specific compared with stem-specific Tfh cells, irrespective of the surface marker combinations used to define antigen-specific Tfh cells (*P* = 0.005 for OX-40^++^ICOS^++^ and OX-40^++^CD25^+^ responses, *P* = 0.008 for CD154^+^ responses) (Figure 4B). We observed similar results in BALB/c mice (Supplemental Figure 15A). Importantly, despite the induction of stem-specific serum antibodies and expansion of Tfh cells in the draining lymph nodes, stem KLH vaccination did not elicit a stem-specific Tfh cell response in C57BL/6 mice (Figure 4C) or in BALB/c mice (Supplemental Figure 15B). We confirmed, using either whole KLH protein or a subset of immunogenic KLH peptides, that robust KLH-specific Tfh responses could be detected in stem KLH–vaccinated animals (Supplemental Figure 16). Together, these results suggest that deficient Tfh cell elicitation may underpin the poor immunogenic potential of the stem-based immunogen.

Extending these findings, we examined the frequency of HA head– and HA stem–specific Tfh cells in the MLNs of mice following intranasal PR8 infection. Consistent with the immunization data, HA head–specific Tfh responses were readily detectible on day 14 after infection, while stem-specific Tfh cells were rarely identified (Figure 4D), suggesting that restricted Tfh cell responses targeting the HA stem are independent of the HA antigen delivery modality.

### Selective recall of stem antibody responses is greatest in the context of HA-FL immunization.

**Figure 5:**
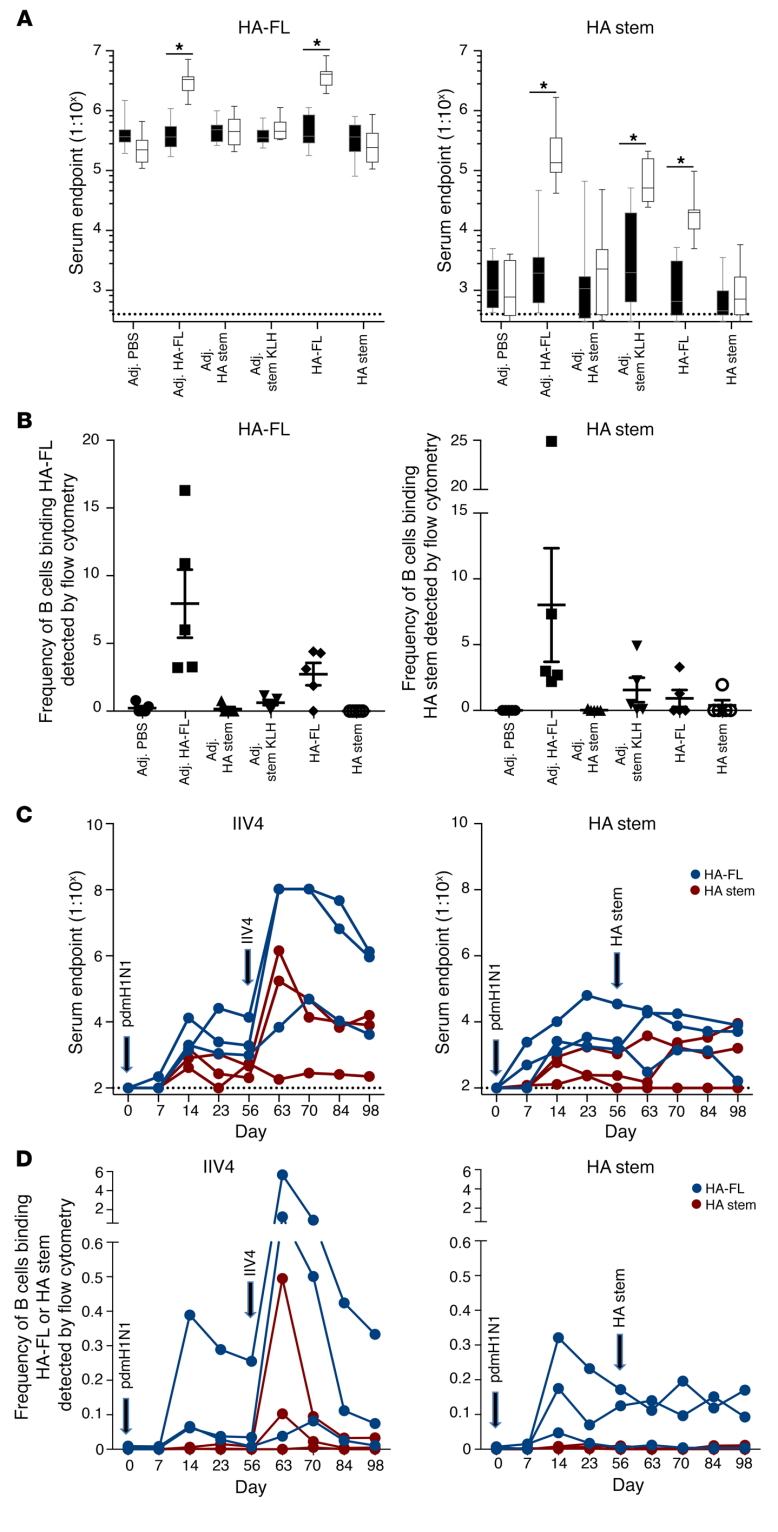
Stem immunogens fail to selectively recall stem antibodies in preimmune mice and macaques. Mice infected intranasally with PR8 and immunized on day 56 were analyzed for (**A**) serum endpoint total IgG titers measured by ELISA on day 56 (black box shows before immunization) and day 70 (white box shows 2 weeks after immunization) using HA-FL or HA stem proteins (*n* = 10 from 2 independent experiments with groups of 5 animals) and (**B**) frequency of GC B cells (B220^+^IgD^–^CD38^lo^GL7^+^) stained with HA-FL and HA stem probes (PR8) measured by flow cytometry (*n* = 5). Box boundaries represent the 25^th^ and 75^th^ percentiles, the inner line represents the median, and whiskers represent minimum and maximum values. Data indicate the mean ± SEM. *P < 0.05, by Mann-Whitney *U* test. Macaques (*n* = 6) infected intranasally with A/Auckland/1/2009 and immunized on day 56 with seasonal IIV4 or HA stem immunogen were analyzed for (**C**) serum endpoint total IgG titers measured by ELISA using CA09 HA-FL (blue) or stabilized CA09 HA stem (red) proteins and (**D**) frequency of IgG^+^ memory B cells (CD19^+^IgD^–^IgG^+^) binding CA09 HA-FL (blue) or stabilized CA09 HA stem (red) probes measured by flow cytometry within cryopreserved PBMC samples. Dotted lines denote the detection cutoff (dilution 1:100).

We next examined the capacity of HA stem immunogens to recall anti-stem antibody responses in the context of preexisting immunity, such as might be found in immune adults. C57BL/6 mice were infected intranasally with PR8 before intramuscular injection 56 days later with HA-FL and HA stem immunogens. High serum antibody titers against HA-FL could be detected after infection in all animals, with a minor boost observed in groups immunized with HA-FL protein with or without adjuvant (Figure 5A). In line with the primary infection model, very low titers of stem-specific serum antibody were observed after infection. However, these stem-specific titers were boosted following immunization with HA-FL or HA stem with a KLH carrier. Interestingly, we found that vaccination with the HA stem immunogen alone, with or without adjuvant, failed to elicit stem antibody responses in these preimmune animals. This was similarly evident when we examined the recruitment of antigen-specific B cells into the draining ILNs, whereby HA-FL efficiently recruited both HA-FL– and stem-specific B cells into GCs following immunization (Figure 5B). KLH-conjugated stem, but not stem alone, recruited stem-specific B cells into GCs.

We next boosted 6 macaques experimentally infected with pdmH1N1 (from Figure 2A) with either 30 μg HA stem protein or a double dose (total of 30 μg of each HA) of the seasonal quadrivalent inactivated influenza vaccine containing a pdmH1N1 component (IIV4; 2016 Fluarix Tetra). Consistent with the mouse model, IIV4 immunization drove the efficient recall of HA-FL (3 of 3 animals) and stem serum antibody responses (2 of 3 animals) (Figure 5C), with a corresponding rise in serum neutralization titers as measured by a focus reduction assay (Supplemental Figure 17). In contrast, immunization with the HA stem (using a conserved H1N1 HA stem immunogen derived from A/New Caledonia/20/1999) (6) failed to recall HA-FL antibody, HA stem antibody, or CA09 serum neutralization activity. These patterns were recapitulated when we examined the frequency of HA-FL– or HA stem–specific B cells in the blood of immunized macaques (Figure 5D), where we observed a boosting of memory B cell frequencies only in the animals that received IIV4. Taken together, our results suggest that the poor immunogenicity of HA stem immunogens observed during primary immunization also translates into a diminished capacity to recall stem-specific memory responses in preimmune animals.

### Seasonal influenza vaccination of humans drives stem-specific antibody and memory B cell expansion in humans.

**Figure 6:**
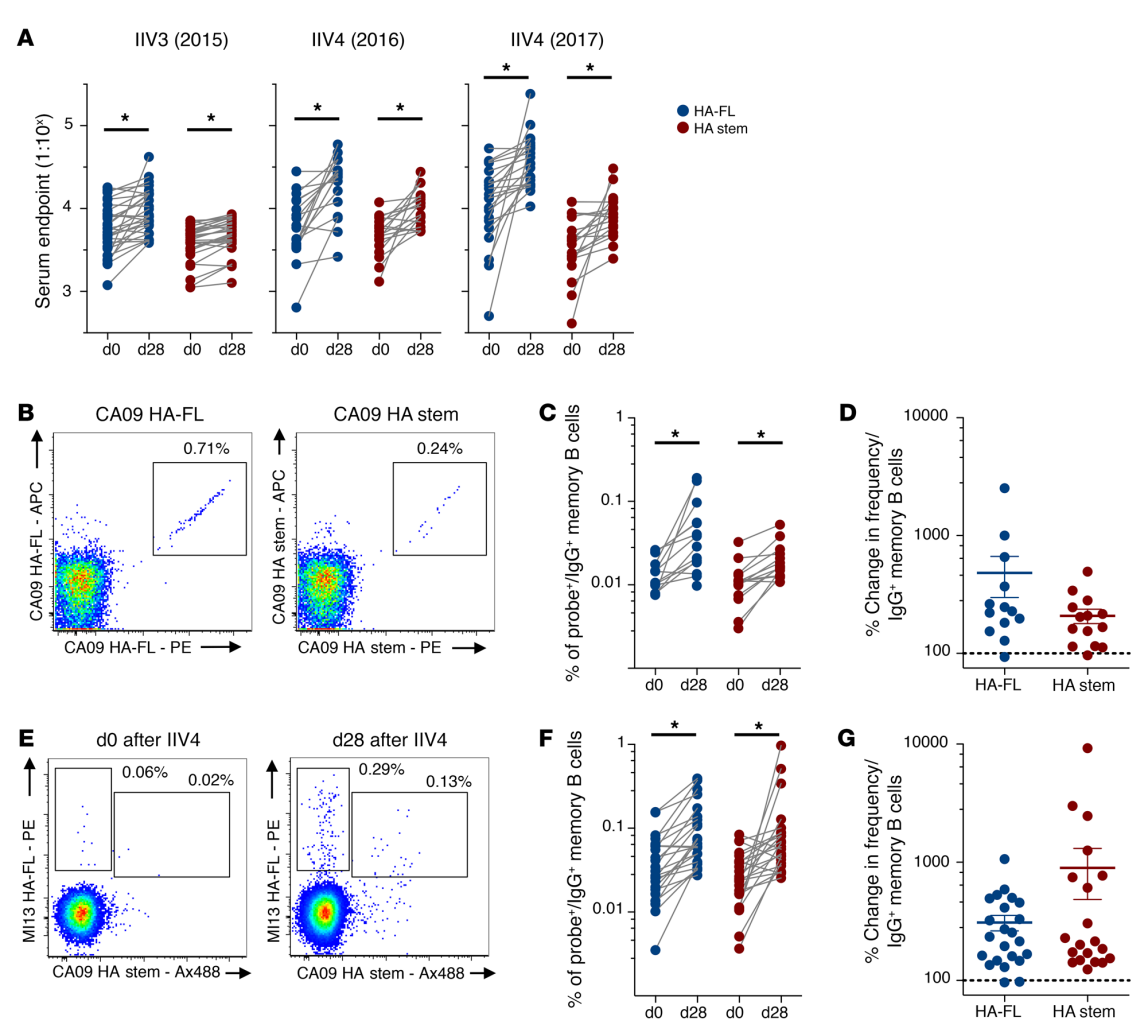
Stem antibody and memory B cells are expanded by seasonal influenza vaccination in humans. (**A**) Serum endpoint total IgG titers of antibody binding the HA-FL (blue) or the stabilized HA stem (red) proteins at baseline and following immunization (day 28 [d28]) with seasonal IIV3 (*n* = 29) or IIV4 (2016: *n* = 18; 2017: *n* = 21). (**B**) Representative flow cytometric plots of IgG^+^ memory B cells from IIV4 (2016 season) recipients double stained with recombinant HA-FL or HA stem probes (CA09). Memory B cells were defined as CD19^+^IgD^–^IgG^+^ after prior exclusion of doublets, dead cells, and CD3^+^, CD14^+^, CD16^+^, CD8^+^, and CD10^+^ cells. Frequency (**C**) and percentage change (**D**) of IgG^+^ memory B cells binding HA-FL or HA stem between baseline and after IIV4 (2016 season) immunization (*n* = 18). (**E**) Representative flow cytometric plots of IgG^+^ memory B cells from IIV4 (2017 season) recipients costained with recombinant HA-FL (A/Michigan/45/2015) or HA stem probes (CA09). Frequency (**F**) and percentage change (**G**) of IgG^+^ memory B cells binding either HA-FL or HA stem between baseline and after IIV4 (2017 season) immunization (*n* = 21). Data represent the mean ± SEM. *P < 0.05, by Mann-Whitney *U* test (**A**, **C**, and **E**).

There have been varying reports as to the degree to which HA stem responses are elicited by seasonal immunization in humans (3, 9, 10). Serological responses to seasonal vaccines were assessed by ELISA in 3 cohorts of healthy Australian adult volunteers receiving Southern Hemisphere formulations of 2015 IIV3 (Fluvax, *n* = 29) (31), 2016 IIV4 (FluQuadri, *n* = 18), or 2017 IIV4 (Afluria Quadrivalent, *n* = 21), all of which contained H1N1 A/California/06/2009 (2015 and 2016 seasons) or the antigenically similar A/Michigan/45/2015 (2017 season). Serum antibody binding HA-FL or HA stem was detected within baseline samples in all subjects, with approximately 2- to 3-fold lower titers of stem-specific antibodies (Figure 6A). We observed a significant expansion of endpoint titers of HA-FL and HA stem antibodies following vaccine administration in all 3 cohorts, but serological titers of stem-specific antibodies were consistently lower than those detected for HA-FL.

Although the use of recombinant trimeric HA probes for ex vivo identification of HA-specific B cells by flow cytometry is well established (2, 11, 21), stem-specific B cell responses to seasonal influenza vaccines have not been extensively characterized. We enumerated memory B cells recognizing HA-FL or the HA stem in seasonal vaccine recipients. Cryopreserved PBMC samples from the IIV4 (2016 season) cohort were costained with a B cell phenotyping panel (gating in Supplemental Figure 18) and HA-FL or HA stem probes, with HA-specific B cell populations double stained to maximize specificity (Figure 6B). Four weeks after immunization, we observed a significant expansion in memory B cell frequencies from baseline for both HA-FL– and stem-specific populations (Figure 6C), with similar overall magnitudes of memory B cell expansion for both cell populations (Figure 6D). Likewise, using samples from the subsequent year’s cohort (IIV4 2017) and costaining with HA-FL and HA stem probes (Figure 6E), we again observed a significant and comparable expansion of HA-specific B cells binding both nonstem and stem regions (Figure 6, F and G). Indeed, a dramatic expansion of stem-specific memory B cells could be directly observed in a subset of vaccine recipients (4 of 21) following immunization (Supplemental Figure 19). To summarize, in subjects with preexisting H1N1 influenza immunity, we confirmed that stem responses are subdominant to head responses but that seasonal influenza vaccines can drive expansion of HA stem–specific humoral immunity. Thus, a general lack of stem-specific B cell responsiveness does not seem to underpin the serological subdominance of stem-specific antibody responses in humans.

## Discussion

There is significant interest in universal influenza vaccination based on antibody responses to the HA stem. Consistent with previous reports (15, 16), we found that in both mice and monkeys, the HA stem was subdominant to antibody responses targeting canonical epitopes in the globular head domain. Stem-specific B cells failed to undergo significant expansion, recruitment to GCs, or differentiation into bone marrow–resident plasma cells following infection. The autoreactivity of human stem antibodies was previously suggested as a potential contributing factor to stem subdominance (13). While autoreactivity or low naive precursor frequencies would likely inhibit the recruitment of stem-specific B cells into a primary response, we found that immunization with recombinant HA-FL, HA stem nanoparticles, or stem KLH conjugates could each induce high stem-specific serum antibody titers. Thus, any stem-specific B cell defects are not absolute, and other factors must contribute to stem subdominance.

The subdominance of the HA stem might reflect an inability of stem-specific B cells to interact with their cognate antigen in vivo. Steric hindrance of neutralizing stem epitopes in the context of whole influenza virions has been previously reported using monoclonal antibodies (13). In the current study, we found that the HA stem antigen was widely prevalent at the site of infection. However, the extent and conformational integrity of HA stem antigens making it to the draining lymph nodes remain unclear. Interestingly, we observed that HA stem subdominance was greatest following viral infection compared with soluble protein vaccination, suggesting that anchoring HA to whole virions does limit B cell accessibility to the stem in vivo. Nevertheless, HA stem subdominance was still evident in the context of soluble protein immunization, in which these steric constraints are minimal.

Vaccines encompassing “headless” or “stabilized” HA stem domains have been developed and shown protection from influenza virus challenge in naive animal models (6, 7, 32–36). Here, we found that repeated immunization of C57BL/6 mice with a stable, trimeric HA stem immunogen, either alone or with adjuvant, elicited markedly reduced serum antibody responses compared with HA-FL or a control PE protein. While the HA-FL immunogen used in this study carried a mutation that abolished binding to cell-surface sialic acids (21), we found that the immunogenicity of the HA protein was not markedly compromised by this change and was broadly comparable to that of the PE control. Indeed, we detected greater stem-specific serum antibody levels in mice immunized with HA-FL compared with those treated with the HA stem in isolation, suggesting that the impact of the HA-FL Y98F mutation on stem responses was minimal. In contrast to the HA stem used in this study, several previously designed HA stem constructs have shown robust immunogenicity and protection in vivo, despite the fact that some variants displayed conformational misfolding (7, 33, 34, 36). Although a comprehensive head-to-head comparison is difficult, these varied reports suggest that any immunogenicity defects identified in the present study were not absolute and were heavily influenced by immunogen design, particularly with substitutions in segments of the HA protein retained, and by the animal system used to evaluate the stem vaccines. Indeed, we found that the different mouse strain used in the current study mounted a better HA stem immunity compared with the C57BL/6 mouse model. Nevertheless, after infection or immunization in all animal models, we saw consistently poorer humoral responses targeting the HA stem compared with HA-FL. The poor immunogenicity of the HA stem was associated with an inability to efficiently elicit CD4 help in vivo, with maximal humoral responses rescued by conjugation to the HA head domain or KLH, both of which successfully induced a Tfh cell response.

A diminished stem-specific Tfh cell response was also observed during infection, suggesting that the HA stem could conceivably be lacking in MHC class II–restricted T cell epitopes relative to the globular head. Although a poor stem-specific Tfh cell response cannot fully explain the subdominance of the stem during infection or in response to vaccination in outbred populations, the murine data suggest that the magnitude of the Tfh cell response could be a limiting factor in determining stem immunogenicity. Consequently, the ability of the immunizing antigen to elicit CD4^+^ T cell help in the context of diverse MHC class II alleles should be an important consideration in the design of stem-based universal influenza vaccines. Intriguingly, while immunodominance of HA-FL over HA stem was clearly observed in infected or vaccinated BALB/c mice carrying distinct MHC-II alleles, we found that HA stem vaccination could elicit a greater stem-specific antibody response than that observed in C57BL/6 mice. In silico epitope analysis predicted 2 putative epitopes of the HA stem domain restricted by the MHC-II molecule in BALB/c mice, while no epitopes were predicted for C57BL/6 mice (data not shown), suggesting that differences in HA stem immunogenicity across these mouse strains may be modulated by the availability of epitopes presented by MHC-II molecules and the resulting induced Tfh cell response. It is also possible that the HA stem and head domains could be differentially susceptible to proteolytic degradation in vivo, which may affect the direct recognition by B cells as well as the limiting peptide substrate presentable to T cells. While we observed that both HA head and HA stem epitopes are bioavailable in the lungs following infection, the integrity and conformational state of HA as presented to B cells in vivo are not known. Further studies are required to accurately characterize the nature of HA localized within the relevant lymph nodes following infection and immunization.

Stem-based universal vaccines need not induce primary B cell responses and instead could target preexisting immunological memory, which in humans originates from near-ubiquitous childhood infection (37, 38). Using infection in mice to establish baseline immunity, we found that HA stem immunogens, even in the presence of an adjuvant, poorly recalled stem memory responses. By contrast, boosting with the HA-FL or KLH-conjugated stem immunogens effectively recalled stem serum antibodies. Similarly, we observed poor recall of the stem response by the HA stem immunogen in macaques with preexisting immunity, while IIV4 vaccination (analogous to HA-FL) led to boosted stem responses. However, our study does not exclude the possibility of enhancing the HA stem immunization when formulated with other adjuvant modalities in macaques. These observations suggest that the poor elicitation of Tfh cells observed during primary immunization and infection might similarly constrain the capacity of stem immunogens to recall immune memory.

HA stem–specific memory B cells have been widely reported in adult humans (10, 11, 39) and were similarly evident within our cohorts. Following immunization with seasonal inactivated influenza vaccines, we observed a consistent expansion of stem-specific immunity at serological and B cell levels, albeit to a lesser magnitude than for non-stem–localized epitopes, broadly consistent with previous reports (3, 10). While stem-specific memory B cells are clearly targetable by vaccination in humans and in preimmune animal models, the longevity of such responses remains unclear, with suggestions that only stem immunity elicited by infection is long lived (9). Further studies into strategies to extend the durability of stem-specific immunity are warranted.

In summary, we found that the immunological dynamics of humoral immunity targeting the HA stem domain are complex and context dependent. Our results suggest that fundamental constraints exist to limit the immunogenicity of the HA stem domain during both infection and immunization, a finding consistent with the broad, evolutionarily conserved immunological subdominance of this region. We cannot rule out the idea that different HA stem–directed vaccine approaches may have differing immunogenic potential, given the diverse range of immunogen designs, expression systems, formulations, immunization schedules, and animal models described (40). It does seem likely, however, that boosting the immunogenicity of the HA stem via novel adjuvants, carrier proteins, or nanoparticle formulations will be necessary to elicit robust titers of stem immunity in humans. Alternatively, strategies that maintain the coupling of head and stem domains might be favorable (41, 42). A greater mechanistic understanding of the molecular basis of immunogenicity and how HA immunodominance hierarchies are established and maintained is required to guide the design of improved vaccination regimes for broad protection against seasonal and emergent influenza viruses.

## Methods

### Seasonal IIV3 and IIV4 clinical samples.

The 2015 IIV3 immunization trial is fully described elsewhere (31) and registered as NCT02632578 (http://www.clinicaltrials.gov). Briefly, 30 healthy Australian adults were vaccinated with the 2015 Fluvax (bioCSL) containing 15 μg HA from A/California/07/2009-like (pdmH1N1), A/Switzerland/9715293/2013 (H3N2)-like, and B/Phuket/3073/2013-like strains. For the 2016 IIV4 trial, 20 healthy adults were administered FluQuadri vaccine (Sanofi) containing A/California/07/2009-like virus (pdmH1N1), A/Hong Kong/4801/2014–like virus (H3N2), B/Phuket/3073/2013-like virus, and B/Brisbane/60/2008-like virus components. For the 2017 IIV4 trial, 22 healthy adults were administered Afluria Quadrivalent (Seqirus) vaccine containing A/Michigan/45/2015 (pdmH1N1), A/Hong Kong/4801/2014-like virus (H3N2), B/Phuket/3073/2013-like virus, and B/Brisbane/60/2008-like virus components. For all trials, sera, plasma, and PBMCs were collected and cryopreserved at baseline (day 0) and on day 28.

### Animal infection and immunization.

For murine trials, C57BL/6 or BALB/c mice at 6 to 8 weeks of age were used. Mice were anesthetized by isoflurane inhalation prior to infection or immunization. For intranasal infections, mice were instilled with a 50-μl volume of fifty 50% tissue culture infective doses (TCID_50_) or five hundred TCID_50_ of PR8 or CA09, respectively. For vaccination studies, proteins were formulated at 5 μg HA or the HA equivalent (stem KLH) diluted in PBS with or without adjuvant (1:1 Addavax, InvivoGen; 1:1 R848, InvivoGen; 1:4 CpG ODN 1826, InvivoGen; 1:1 complete/incomplete Freund’s adjuvant, MilliporeSigma; 1:1 Sigma Adjuvant System, MilliporeSigma). Vaccinations were administered intramuscularly into both hind quadriceps or subcutaneously into the abdomen.

Eight influenza-naive juvenile pigtail macaques (*Macaca nemestrina*) were used for the study. Prior to any procedure, the animals were anesthetized intramuscularly with ketamine. The macaques were inoculated with 1 × 10^7^ PFU A/Auckland/1/2009 via the larynx and tonsils as previously described (17), and serial blood samples were subsequently obtained, with cryopreservation of PBMCs and serum. Animals were immunized via both hind quadriceps with 2 doses of the 2016 Southern hemisphere IIV4 vaccines (GSK Fluarix Tetra) or, alternatively, with 30 μg H1N1-stabilized stem protein derived from A/New Caledonia/20/1999 (6).

### HA proteins.

Recombinant HA-FL proteins used in immunizations, ELISA, and flow cytometric assays were derived for PR8, A/California/07/2009, and A/Michigan/45/2015 as previously described (21). HA-FL proteins carry a Y98F mutation in the receptor-binding site, which abolishes binding to cell-surface sialic acids. Stabilized HA stem proteins were engineered for PR8, A/New Caledonia/20/1999, and A/California/07/2009 using methods established previously for the design of Gen6 HA stem in Yassine et al. (6). Briefly, expression constructs were synthesized (GeneArt) and cloned into mammalian expression vectors. HA-FL and HA stem proteins were expressed by transient transfection of Expi293 (Life Technologies, Thermo Fisher Scientific) suspension cultures and purified by polyhistadine-tag affinity chromatography and gel filtration. Proteins were biotinylated using BirA (Avidity) and stored at –80°C. Prior to use, biotinylated HA proteins were labeled by the sequential addition of streptavidin (SA) conjugated to PE, allophycocyanin (APC), or BV421 and stored at 4°C.

### Flow cytometric detection of HA-specific B cells.

HA-specific B cells were identified within cryopreserved human PBMCs by costaining with HA probes conjugated to SA-PE, SA-APC, SA-BV421, or SA-Ax488 (all from BD). Cells were stained with Aqua Viability Dye (Thermo Fisher Scientific). The monoclonal antibodies used for surface staining included the following: CD19-ECD (J3-119) (Beckman Coulter); CD20 Alexa 700 (2H7), IgM-BUV395 (G20-127), CD21-BUV737 (B-ly4), IgD PE-Cy7 (IA6-2), and IgG-BV786 (G18-145) (all from BD); CD14-BV510 (M5E2), CD3-BV510 (OKT3), CD8a-BV510 (RPA-T8), CD16-BV510 (3G8), CD10-BV510 (HI10a), and CD27-BV605 (O323) (all from BioLegend). Background B cells interacting with SA were excluded by staining with SA-BV510 (BD). For macaque samples, PBMCs were similarly stained with HA probes and the human surface panel with the following alterations: IgD–Alexa 488 (poly; Southern Biotech), CD45 PE-Cy7 (D058-1283; BD), and CD20 BUV737 (2H7; BD). For murine samples, tissues were mechanically homogenized into single-cell suspensions in RF10 media (RPMI 1640, 10% FCS, 1× penicillin-streptomycin-glutamine; Life Technologies, Thermo Fisher Scientific). For bone marrow samples, cells were recovered by perfusion of both femurs with RF10. RBC lysis was performed with Pharm Lyse (BD). Isolated cells were stained with Aqua Viability Dye (Thermo Fisher Scientific) and Fc blocked with an anti-CD16/32 antibody (clone 93; BioLegend). Cells were then surface stained with the relevant HA probes and the following antibodies: B220 BUV737 (RA3-6B2; BD); IgD BUV395 (11-26c.2a; BD); CD45 Cy7APC (30-F11; BD); GL7 Alexa 488 (GL7; BioLegend); CD38 PE-Cy7 (clone 90; BioLegend); SA BV786 (BD); CD3 BV786 (145-2C11; BioLegend); and F4/80 BV786 (BM; BioLegend). Bone marrow samples were surface stained with the aforementioned panel and CD138 BB515 (MI-15; BD) and CD138 BV711 (MI-15; BD) and then stained intracellularly with HA probes following fixation and permeabilization. Cells were washed twice, fixed with 1% formaldehyde (Polysciences), and acquired on a BD LSR Fortessa using BD FACSDiva.

### Flow cytometric detection of antigen-specific Tfh cells.

For ex vivo Tfh cell quantification, freshly isolated ILN single-cell suspensions were stained with the following antibodies: Live/Dead Red (Life Technologies, Thermo Fisher Scientific); CD3 BV510 (145-2C11; BioLegend); PD-1 BV786 (29F.1A12; BioLegend); CXCR5 BV421 (L138D7; BioLegend); CD4 BUV737 (RM4-5; BD); ICOS PerCP–eFluor710 (15F9; Life Technologies, Thermo Fisher Scientific); B220 BV605 (RA3-6B2; BD); and F4/80 PE-Dazzle 594 (T45-2342; BD). To identify antigen-specific Tfh cells, freshly isolated ILN, MLN, or splenic cell samples were cultured in RF10 media for 18 hours at 37°C. The samples were stimulated with a peptide pool (2 μg/peptide/ml) comprising the HA head (50 peptides) or HA stem domain (32 peptides) or with a DMSO control. Peptide pools were generated from a PR8 HA peptide array (17 mers overlapping by 11 amino acids, BEI Resources) resuspended in DMSO. To identify KLH-specific Tfh cells, immunogenic 15-mer peptides were predicted by TepiTool (http://tools.iedb.org/tepitool/; Immune Epitope Database and Analysis Resource). The 20 most immunogenic peptide predictions were synthesized (GenScript) and pooled for use in the Tfh assay. In some cases, cells were stimulated with 5 μg/ml whole protein (BSA or KLH). To facilitate protein processing, lymph node suspensions labeled with CellTrace yellow dye (Thermo Fisher Scientific) were cocultured with splenocytes at a 10:1 ratio. At the time of stimulation, an anti–CD154 BV650 or APC mAb (MR1; BioLegend and BD, respectively) was added to all culture conditions. After stimulation, cells were washed twice in PBS and stained with Red Viability Dye (Life Technologies, Thermo Fisher Scientific) according to the manufacturer’s instructions. Cells were then stained with CD3 BV510 (145-2C11; BioLegend); CD25 BB515 (PD61; BD); PD-1 BV786 (29F.1A12; BioLegend); CXCR5 BV421 (L138D7; BioLegend); CD4 BUV737 (RM4-5; BD); OX-40 PeCy7 (OX-86; BioLegend); ICOS PerCP–eFluor 710 (15F9; Life Technologies, Thermo Fisher Scientific); B220 BV605 (RA3-6B2; BD); and F4/80 PE-Dazzle 594 (T45-2342; BD) before being washed, fixed, and acquired on a BD LSR Fortessa using BD FACSDiva.

### ELISA.

Antibody binding to HA-FL or HA stem proteins was tested by ELISA. For human samples, 96-well ImmunoSorp plates (Thermo Fisher Scientific) were coated overnight at 4°C with 2 μg/ml recombinant HA proteins. After blocking with 1% FCS in PBS, duplicate wells of serially diluted serum were added and incubated for 2 hours at room temperature. Plates were washed prior to incubation with a 1:30,000 dilution of HRP-conjugated anti–human IgG (MilliporeSigma) for 1 hour at room temperature. For macaque samples, the plates were coated overnight at 4°C with 2 μg/ml recombinant HA proteins and blocked with 5% BSA in PBS. Duplicate wells of serially diluted serum were added and incubated for 2 hours at room temperature. The plates were washed prior to incubation with a 1:10,000 dilution of HRP-conjugated anti–monkey IgG (Rockland) for 1 hour at room temperature. For murine samples, the plates were coated overnight at 4°C with 2 μg/ml recombinant HA proteins and blocked with 5% BSA in PBS. Duplicate wells of serially diluted serum were added and incubated for 2 hours at room temperature. The plates were washed prior to incubation with a 1:10,000 dilution of HRP-conjugated anti–mouse IgG (KPL) for 1 hour at room temperature. In all cases, the plates were washed and developed using tetramethylbenzidine (TMB) substrate (MilliporeSigma) and read at 450 nm. Endpoint titers were calculated as the reciprocal serum dilution giving signal 2× background using a fitted curve (4-parameter log regression).

### Confocal microscopy.

Fresh tissues were snap-frozen in OCT compound (Sakura Finetek USA) and stored at –80°C. Tissues were sectioned at 7-μm thickness (Leica). Prior to staining, sectioned tissues were fixed in cold acetone solution (MilliporeSigma) for 10 minutes. Tissues were rehydrated with PBS for 10 minutes and blocked with 5% (w/v) BSA (MilliporeSigma) and 2% (v/v) normal goat serum (NGS). To eliminate the background signal of SA probes, endogenous biotin was blocked using a SA/biotin kit according to the manufacturer’s protocol (Life Technologies, Thermo Fisher Scientific).

For in situ staining of influenza-specific B cells, tissues were incubated with 2 μg/ml HA probe conjugated to PE. To amplify the PE signal, tissues were stained sequentially with rabbit polyclonal anti-PE antibodies (Novus Biologicals) and a secondary goat anti–rabbit IgG Alexa Fluor 555 antibody (Life Technologies, Thermo Fisher Scientific). Influenza antigen staining in lungs was performed with 10 μg/ml of the following monoclonal antibodies: 441D6 (anti–HA head, provided by Masaru Kanekiyo from the Vaccine Research Center, NIAID, NIH); CR9114 (anti–HA stem); C179 (anti–HA stem); D1-11 (anti-NP); and VRC01 (noninfluenza control). The monoclonal antibodies CR9114 (1), C179 (43), D1-11 (44) and VRC01 (45) were generated in-house using publicly available sequences. Monoclonal antibody binding was subsequently detected with a secondary goat anti–human IgG Alexa Fluor 555 antibody (Life Technologies, Thermo Fisher Scientific). Cell staining was performed using the following antibodies: B220 (RA3-6B2; BD); GL7 (GL7; BioLegend); and CD35 (8C12; BD). Slides were sealed with ProLong Diamond Antifade Mountant (Life Technologies, Thermo Fisher Scientific). Tiled images were captured on a Zeiss LSM710 instrument. Postprocessing of confocal images was performed with ImageJ, version 2.0.0 (NIH).

### Focus reduction assay.

Neutralization activity of infected macaque sera was assessed against A/Auckland/1/2009 using focus reduction assays as previously described (46). The neutralization titer is expressed as the reciprocal of the highest serum dilution at which virus infection was inhibited by 50% or more.

### Statistics.

Data are generally presented as the median ± IQR or the mean ± SEM. Statistical significance was assessed by Mann-Whitney *U* or Wilcoxon matched-pairs tests. All tests were 2 tailed, and a *P* value of less than 0.05 was considered significant. Curve fitting was performed using 4-parameter logistic regression. Flow data were analyzed with FlowJo, version 9 or 10, and all statistical analyses were performed using GraphPad Prism, version 7 (GraphPad Software).

### Study approval.

The study protocols were approved by the Alfred Hospital Ethics Committee (no. 432/14) and the University of Melbourne Human Research Ethics Committee (no. 1443420), and all associated procedures were performed in accordance with the approved guidelines. Use of the juvenile pigtail macaques (*Macaca nemestrina*) was approved by the Commonwealth Scientific and Industrial Research Organization Animal Health Animal Ethics Committee. All participants provided written informed consent in accordance with Declaration of Helsinki principles. Animal studies and related experimental procedures were approved by the University of Melbourne Animal Ethics Committee (no. 1714193).

## Author contributions

HXT, SJ, JAJ, SJK, and AKW designed the study. HXT, SJ, JAJ, RE, YL, JW, HGK, SJK, and AKW performed experiments. JWY provided key reagents. DT and ACH performed serological and virological analyses. HXT, JAJ, SJK, and AKW wrote the manuscript. All authors read and revised the manuscript.

## Supplementary Material

Supplemental data

## Version 1 12/06/2018

In-Press Preview

## Version 2 01/22/2019

Electronic publication

## Version 3 02/01/2019

Print issue publication
