# Supplementary material for: Subdominance and poor intrinsic immunogenicity limit humoral immunity targeting influenza HA stem
Source: J Clin Invest. 2019 Jan 22;129(2):850–62. doi: 10.1172/JCI123366 (PMC6355240; doi:10.1172/JCI123366)
Supplement: Supplemental data [file jci-129-123366-s279.pdf]

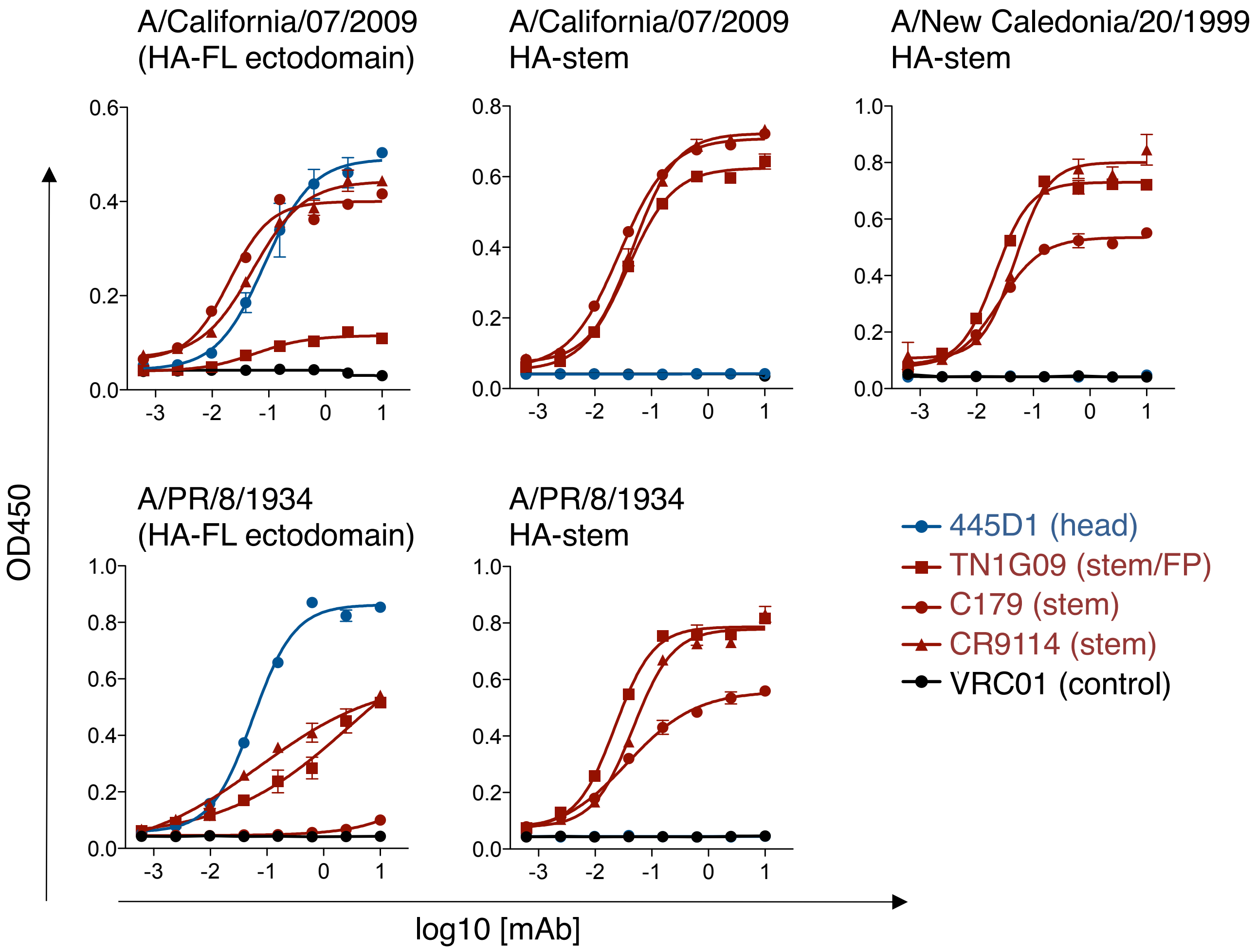

Figure S1. Antigenicity of HA-FL and HA-stem proteins
The antigenicity of full length HA0 ectodomains and stabilised HA-stem proteins was examined by ELISA using a panel of monoclonal antibodies: 445D1 (HA-head specific), TN1G09 (IGHV6-1 class, HA-stem/fusion peptide), C179 (HA-stem), CR9114 (HA-stem) and VRC01 (non-influenza control).

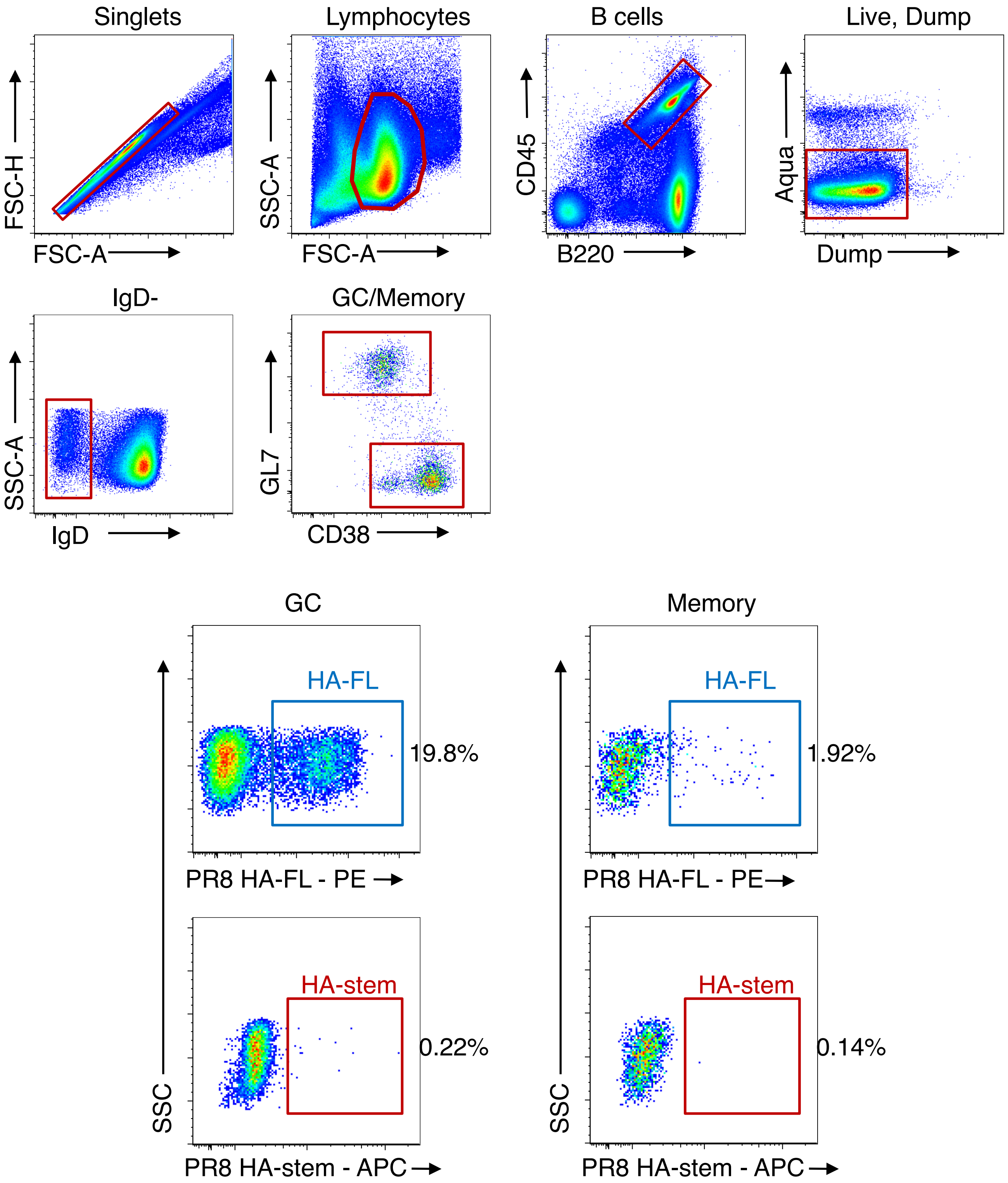

Figure S2. Representative gating for B cell analyses of infected mice
After doublet removal, CD45+B220+IgD- lymphocytes were gated to remove dead and CD3, F4/80 and streptavidin-specific cells before assessing binding to HA-FL or HA-stem probes within germinal centre (GL7+ CD38mid) and memory populations (GL7- CD38hi). Pictured is mediastinal lymph node (MLN) from an infected mouse 28 days after intranasal infection with A/Puerto Rico/08/1934.

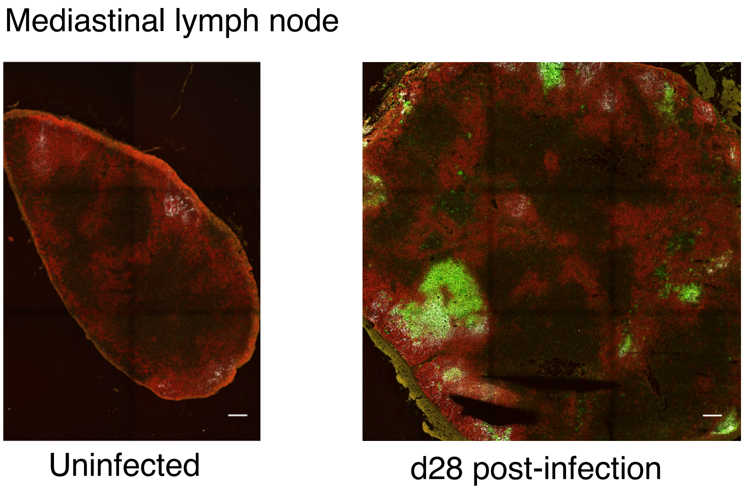

Figure S3. Germinal centre expansion in the mediastinal lymph node following intranasal infection with A/Puerto Rico/08/1934

Tissue sections were stained with CD35 (cyan), GL7 (green) and B220 (red) (scale bar – 100 μm).

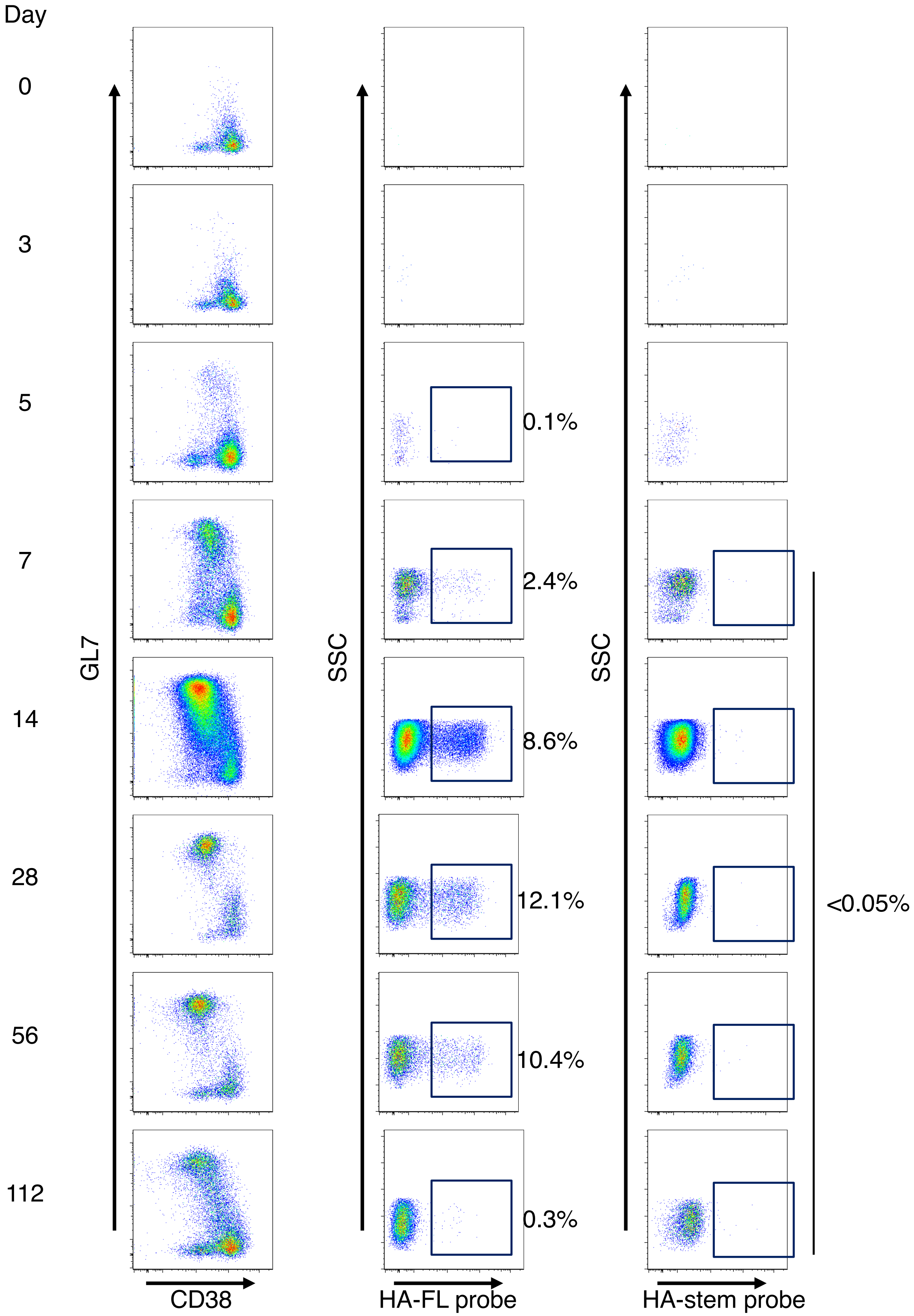

Figure S4. Representative staining of B cell changes in the mediastinal lymph node following intranasal infection with A/Puerto Rico/08/1934. Within expanded GL7+ GC B cells, elevated proportions of HA-FL-specific but not HA-stem-specific B cells can be observed in infected mice.

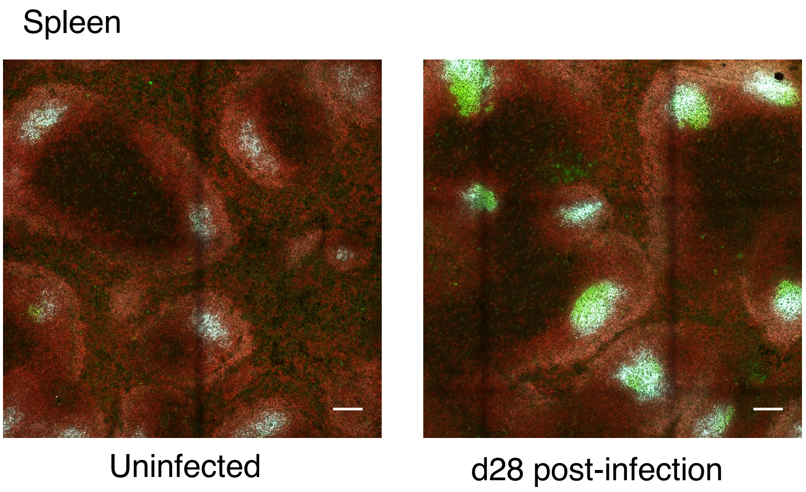

Figure S5. Germinal centre expansion in the spleen following intranasal infection with A/Puerto Rico/08/1934

Tissue sections were stained with CD35 (cyan), GL7 (green) and B220 (red) (scale bar – 100 μm).

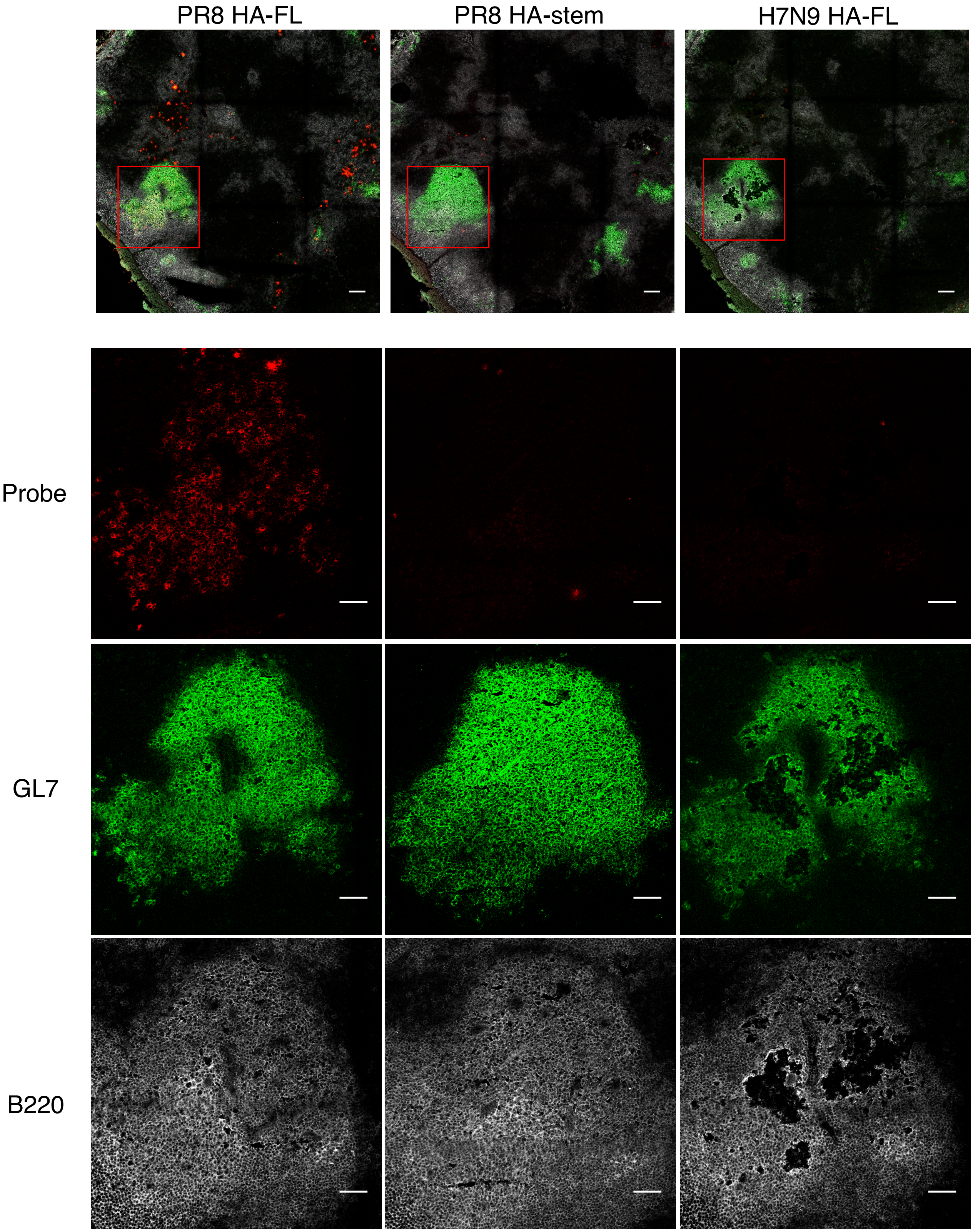

Figure S6. In situ staining for antigen specific B cells in the MLN of infected mice
Tissue sections were stained with HA probe (red), GL7 (green) and B220 (grey) (Gross tissue scale bar – 100 µm; inset GC scale bar – 50 µm).

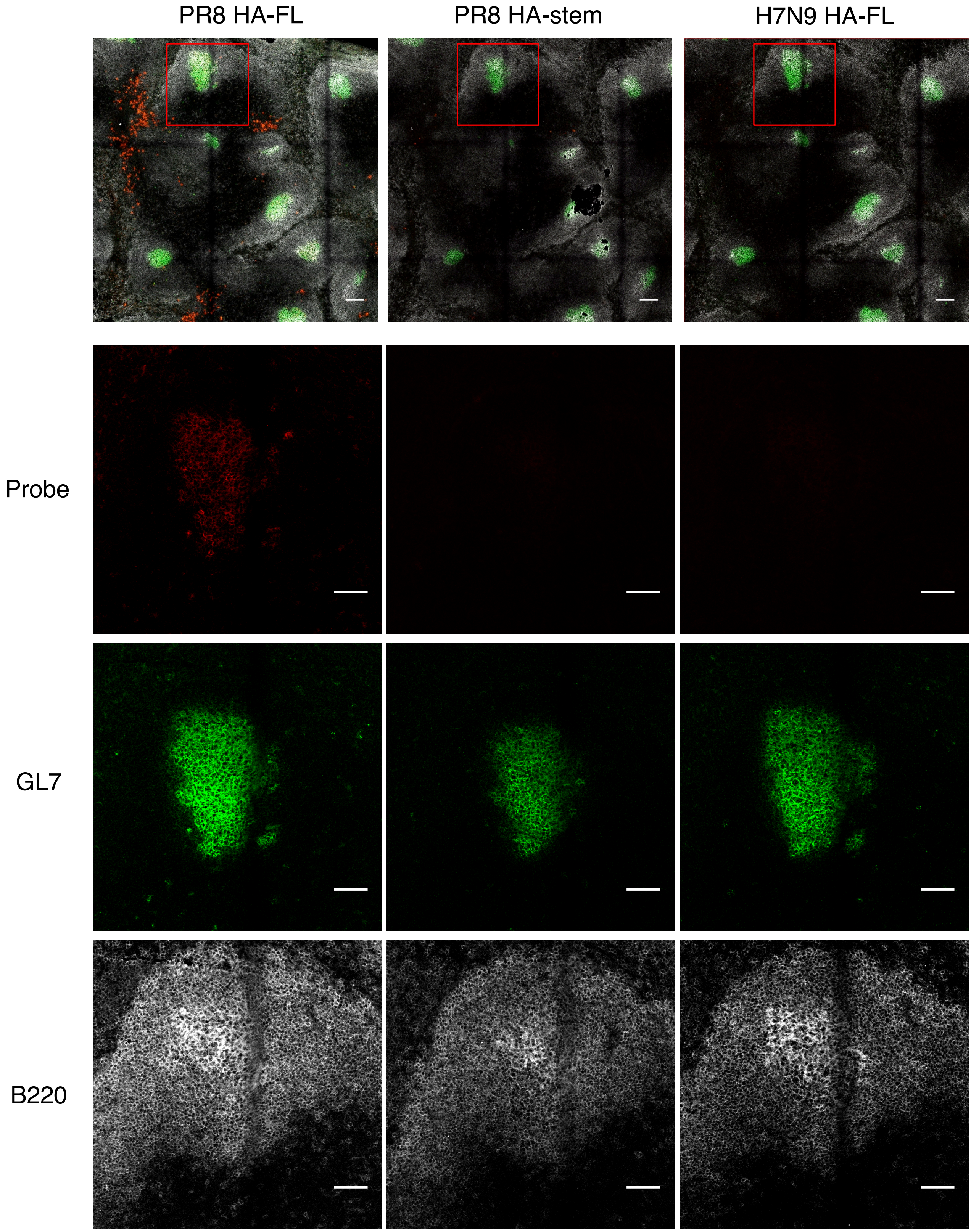

Figure S7. In situ staining for antigen specific B cells in the spleen of infected mice
Tissue sections were stained with HA probe (red), GL7 (green) and B220 (grey) (Gross tissue scale bar – 100 μm; inset GC scale bar – 50 μm).

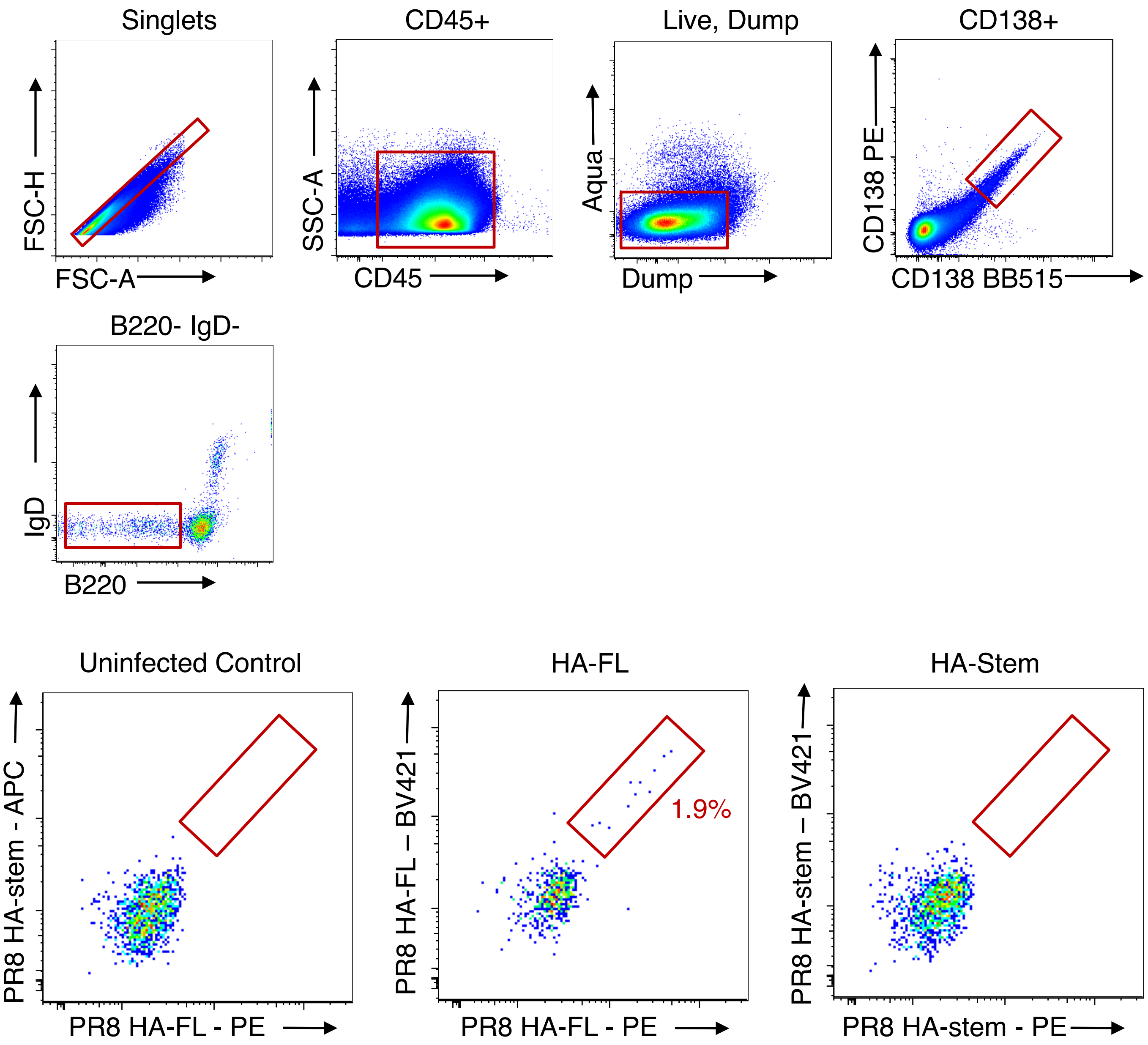

Figure S8. Representative gating for plasma cell analyses of infected mice
CD45+ bone marrow cells were gated to remove doublets, dead, CD3+, F4/80+ and streptavidin-binding cells. Plasma cells were identified based upon double staining for CD138 and negative for B220 and IgD. Intracellular binding to HA-FL or HA-stem probes was assessed. Pictured is an uninfected control and an infected mouse 56 days after intranasal infection with A/Puerto Rico/08/1934.

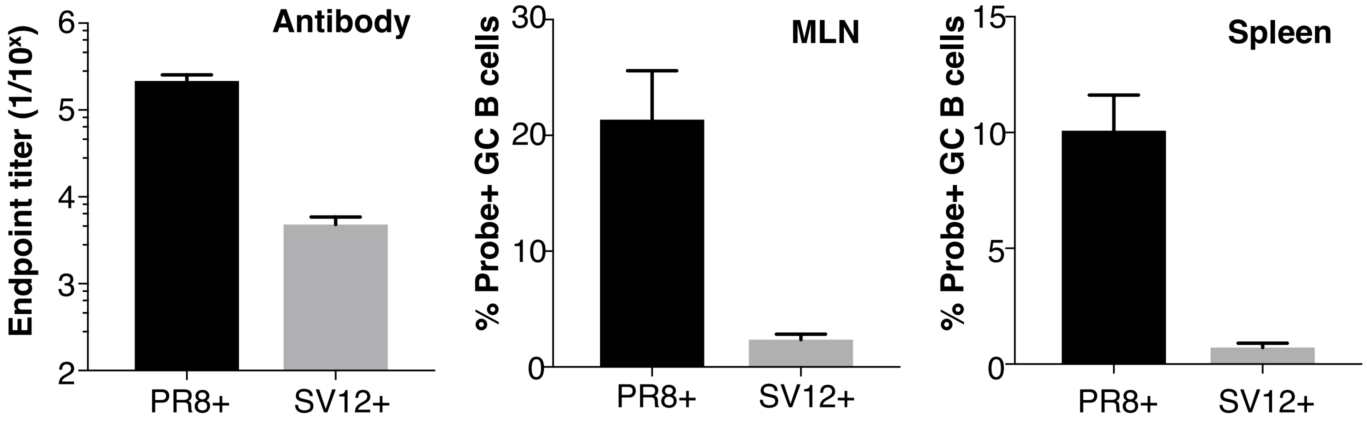

Figure S9. Antibody and B cell responses in PR8 infected mice are focussed upon canonical epitopes within the HA head domain

Antibody and B cells specific for PR8 HA or SV12 HA were measured 14 days post-infection in mice infected intranasally with A/Puerto Rico/08/1934 (n = 5). Serum endpoint IgG titres were determined by ELISA using recombinant PR8 HA or SV12 HA. The proportion of GC B cells (B220+IgD-GL7+CD38lo) binding PR8 or SV12 HA probes within MLNs and spleens of infected mice was assessed by flow cytometry.

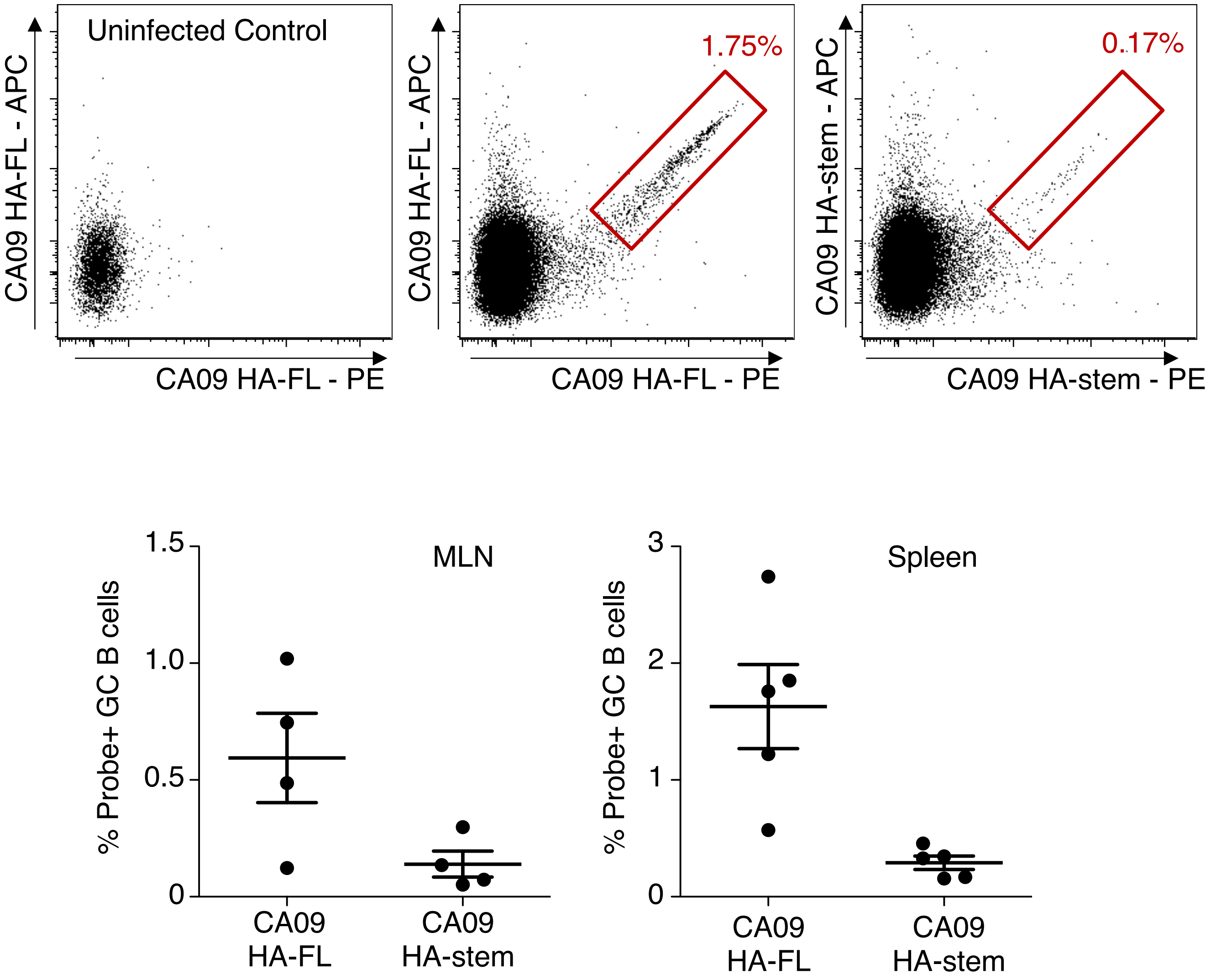

Figure S10. Head- and stem-specific B cells in CA09 infected mice
The proportion of B cells binding HA-FL or the HA-stem probes was assessed in mice infected intranasally with A/California/04/2009 (500 TCID50) (n = 4-5). Spleen and MLNs were harvested 14 days post-infection and the specificity of GC B cells (B220+IgD-GL7+CD38lo) binding HA probes assessed by flow cytometry.

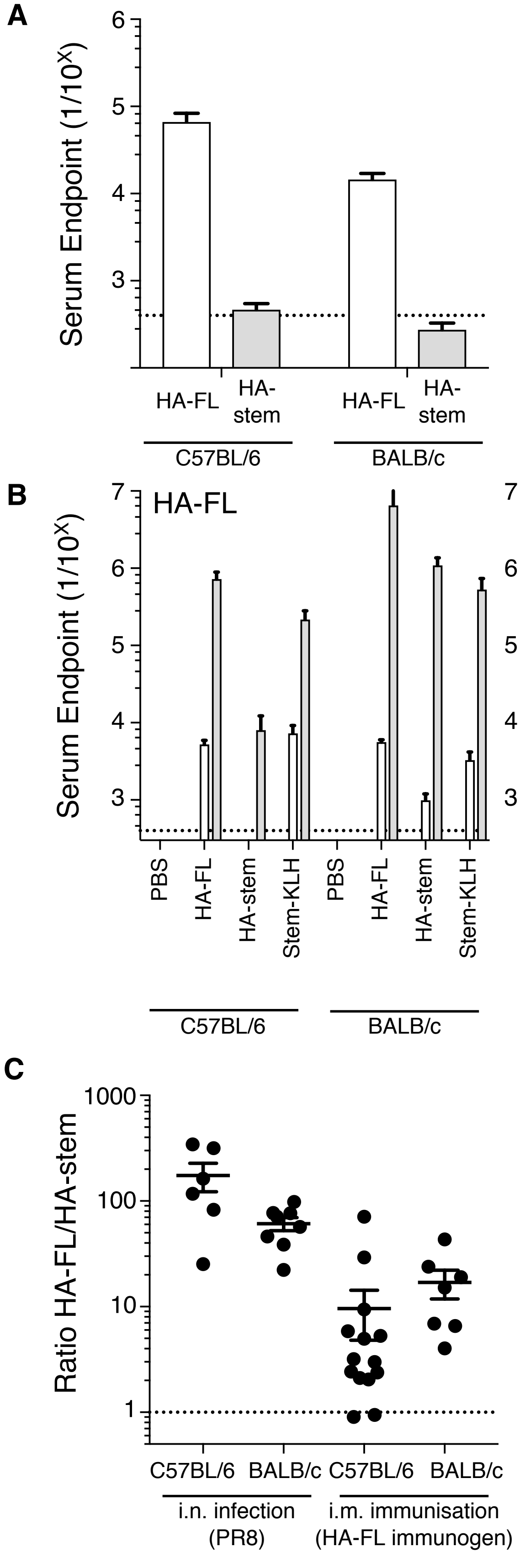

Figure S11. Comparison of serological responses in C57BL/6 and BALB/c mice after vaccination or infection

Serum endpoint total IgG titres were measured by ELISA using HA-FL, HA-stem or KLH proteins in mice that were **(A)** infected intranasally with A/Puerto Rico/08/1934 (n = 6 C57BL/6, n = 8 BALB/c) or **(B)** immunised twice at 3-week intervals with adjuvanted (Addavax) immunogens (n = 5 per group). Serum was analysed 2 weeks post-immunisation or infection. **(C)** HA-FL immunodominance over HA-stem by measurement of serum endpoint IgG titre ratios compiled from infected (n = 6 C57BL/6, n = 8 BALB/c) or HA-FL immunised mice (n = 15 C57BL/6, n = 7 BALB/c). Dotted lines denote detection cut off (dilution 1:400).

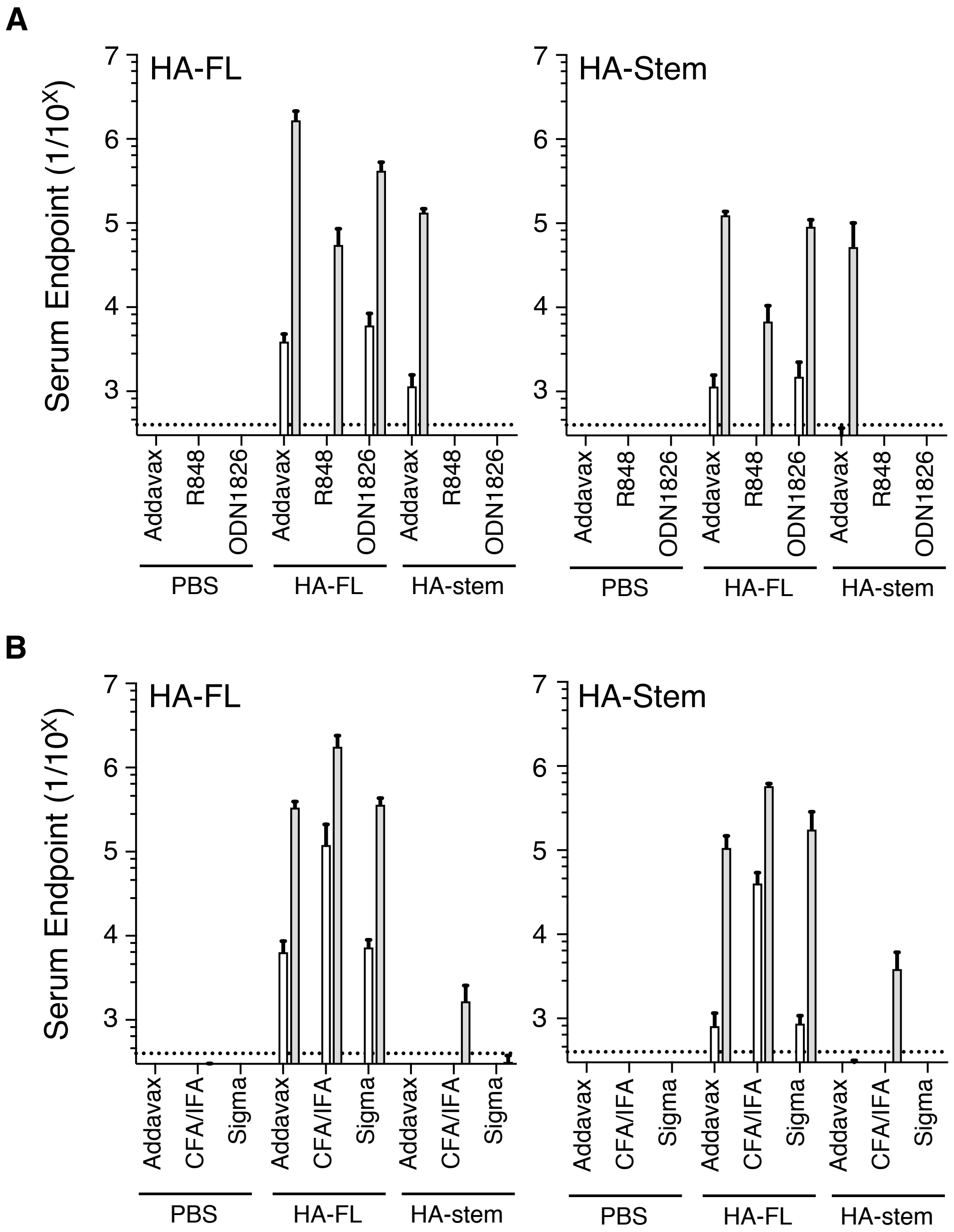

Figure S12. Adjuvants fail to rescue HA-stem antibody responses in C57BL/6 mice
Serum endpoint total IgG titres were measured by ELISA using HA-FL or HA-stem proteins in mice that were immunised **(A)** intramuscularly or **(B)** subcutaneously twice at 3-week intervals with adjuvanted immunogens (n = 5). Serum was analysed 2 weeks post-immunisation. Dotted lines denote detection cut off (dilution 1:400).

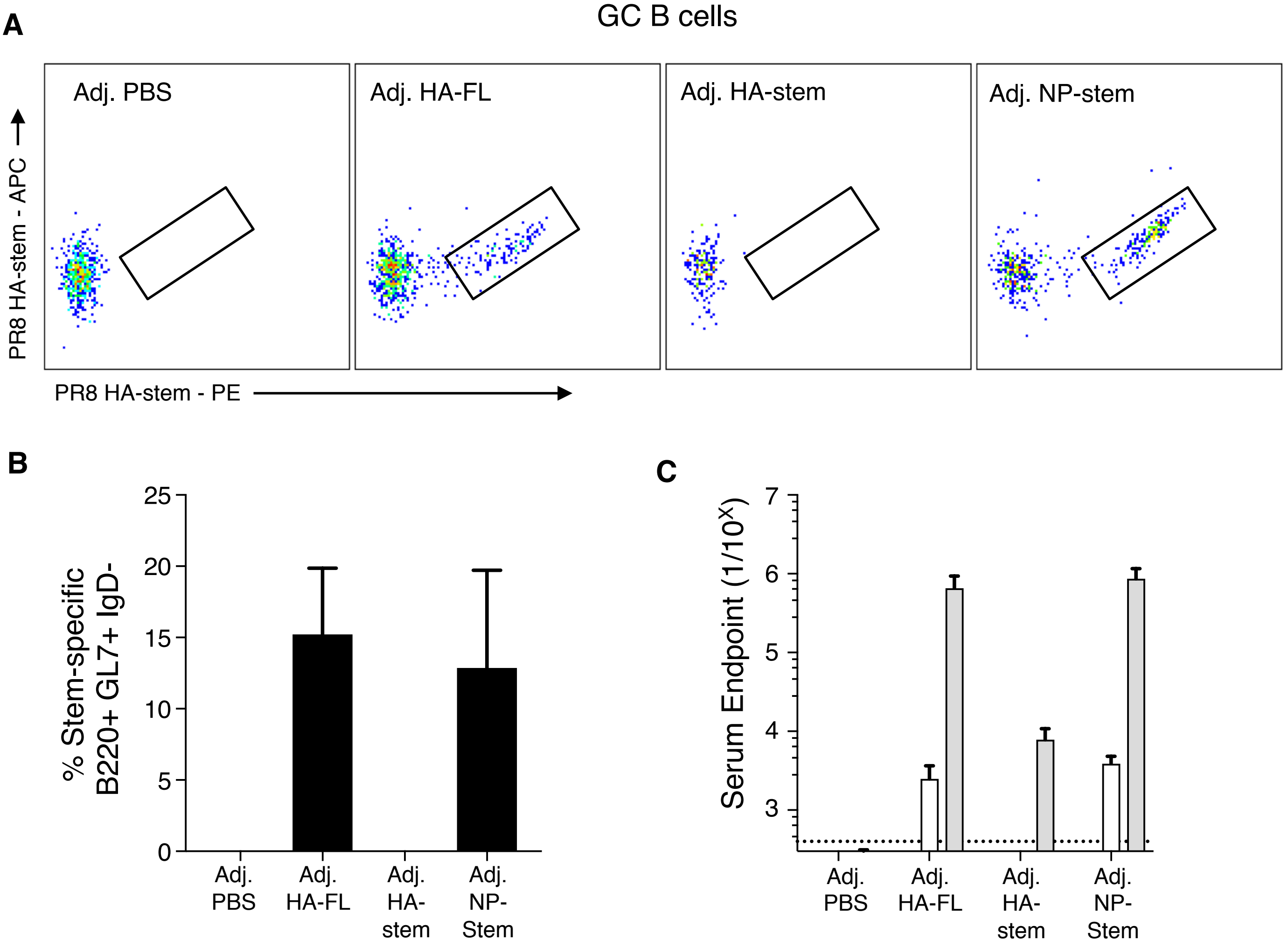

Figure S13. Nanoparticle (NP) carrier rescues B cell and antibody responses against HA-stem

(A) Representative flow cytometry plots and **(B)** frequency of MLN GC B cells (B220+/IgD-/CD38lo/GL7+) double stained with HA-stem probes (A/Puerto Rico/08/1934). **(C)** Serum endpoint total IgG titres were measured by ELISA using HA-stem proteins. Mice were vaccinated twice intramuscularly at 3-week intervals with adjuvanted (Addavax) immunogens (n = 5). B cell probe staining was performed 2 weeks after the last immunisation and serum was analysed every 2 weeks post-immunisation. Dotted lines denote detection cut off (dilution 1:400). Data represent mean ± SEM.

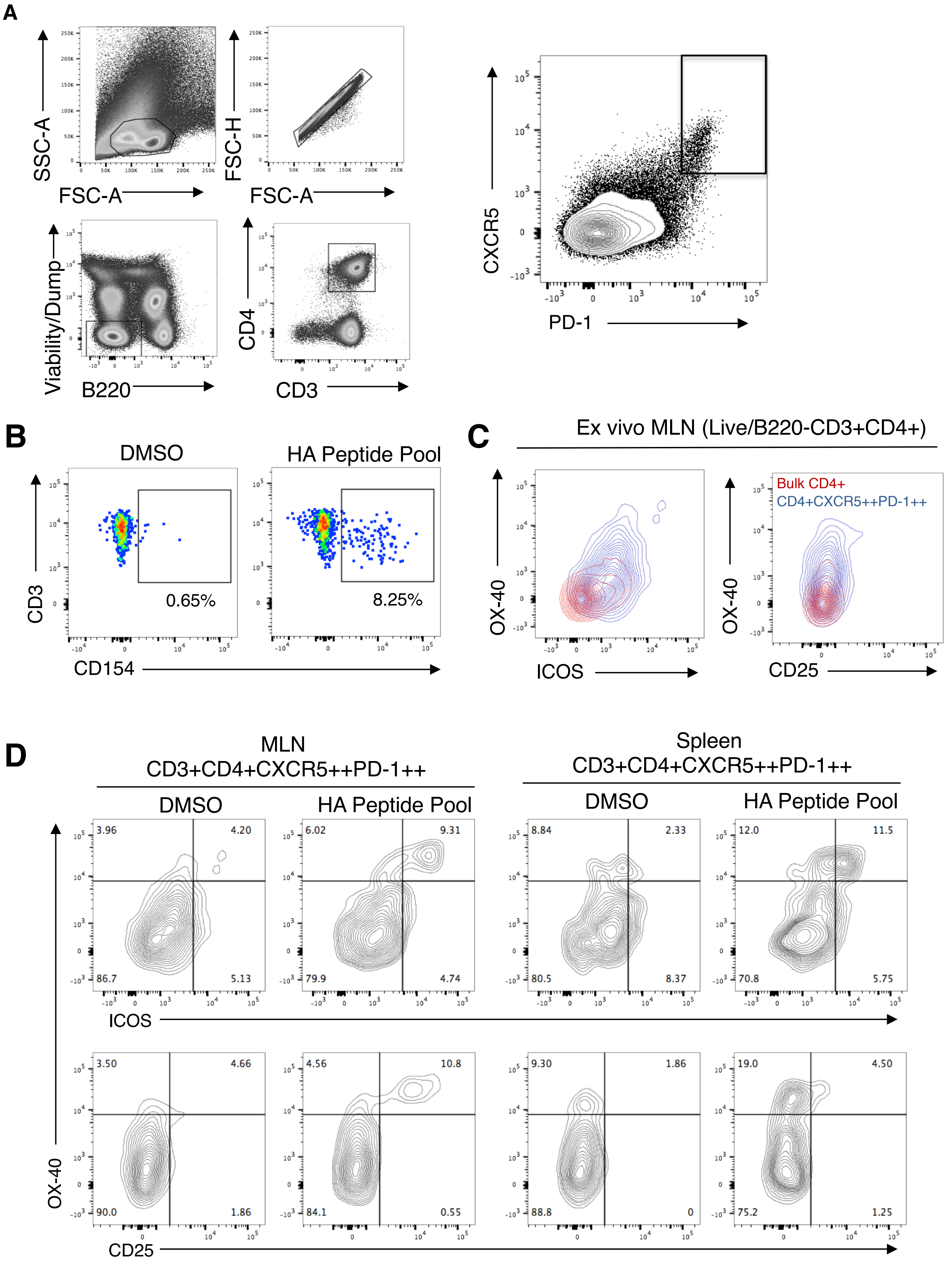
A
SSC-A
FSC-A
FSC-H
FSC-A
CXCR5
PD-1
Viability/Dump
B220
CD4
CD3
B
DMSO
HA Peptide Pool
CD3
0.65%
8.25%
CD154
C
Ex vivo MLN (Live/B220-CD3+CD4+)
OX-40
ICOS
Bulk CD4+
CD4+CXCR5++PD-1++
OX-40
CD25
D
MLN
CD3+CD4+CXCR5++PD-1++
Spleen
CD3+CD4+CXCR5++PD-1++
DMSO
HA Peptide Pool
DMSO
HA Peptide Pool
3.96
4.20
86.7
5.13
6.02
9.31
79.9
4.74
8.84
2.33
80.5
8.37
12.0
11.5
70.8
5.75
ICOS
3.50
4.66
90.0
1.86
4.56
10.8
84.1
0.55
9.30
1.86
88.8
0
19.0
4.50
75.2
1.25
OX-40
CD25

Figure S14. Identification of bulk and antigen-specific Tfh cells in the lymph node and spleen
(A) Bulk Tfh cells were identified first by gating on lymphocytes (FSC-A vs SSC-A) and single cells (FSC-A vs FSC-H). After removal of dead/F4-80+/B220+ cells, CD3+CD4+ T cells were identified and the Tfh population was defined as CXCR5++PD-1++ cells. **(B)** At day 14 post-infection with A/Puerto Rico/08/1934 virus, MLN-derived cells or splenocytes were stimulated with an HA peptide pool in the presence of anti-CD154 antibody to identify antigen-specific upregulation of CD154 compared to a DMSO control. **(C)** Representative ex vivo staining of OX-40, ICOS and CD25 expression on MLN CD4+ T cells and Tfh cells. **(D)** Identification of antigen-specific Tfh cells following 18 hours of peptide stimulation by gating on OX-40++ICOS++ and OX-40++CD25+ cells. Representative staining is shown for MLN and splenocyte-derived Tfh cells.

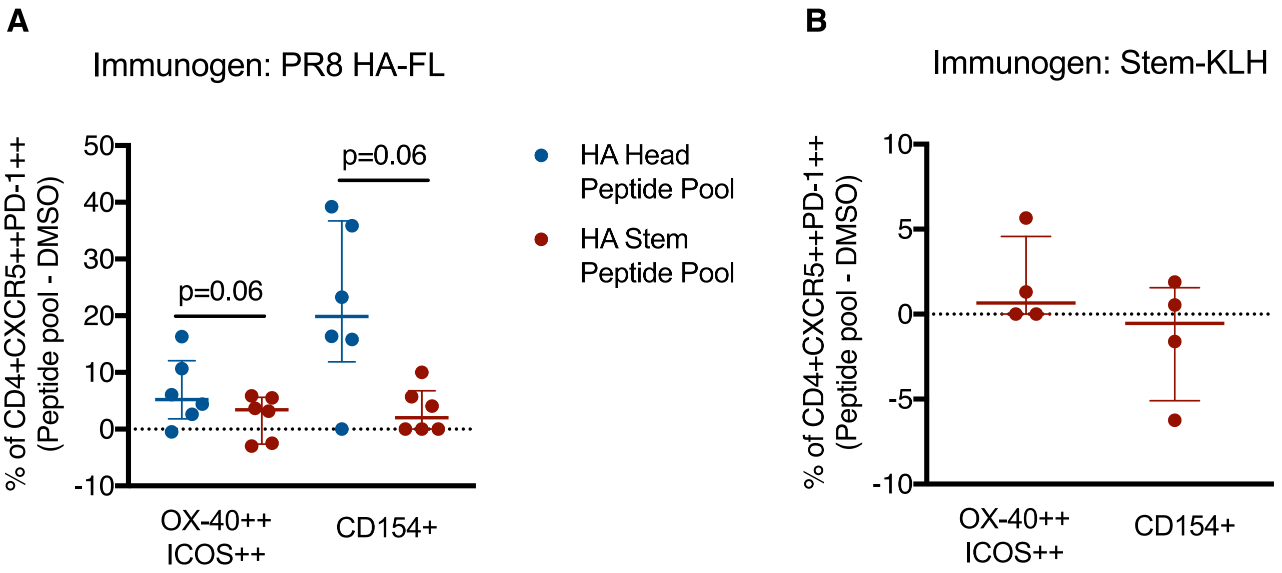

Figure S15. Identification of antigen-specific Tfh cells in BALB/c mice
ILN cells were collected at day 14 post-vaccination with (A) HA-FL protein (n = 6) or (B) stem-KLH protein (n = 4) and stimulated with HA Head (blue dots) or HA Stem (red dots) peptide pools. Antigen-specific Tfh cells were quantified according to surface marker upregulation or CD154 expression as in Figure 4. Bars indicate median and interquartile range.

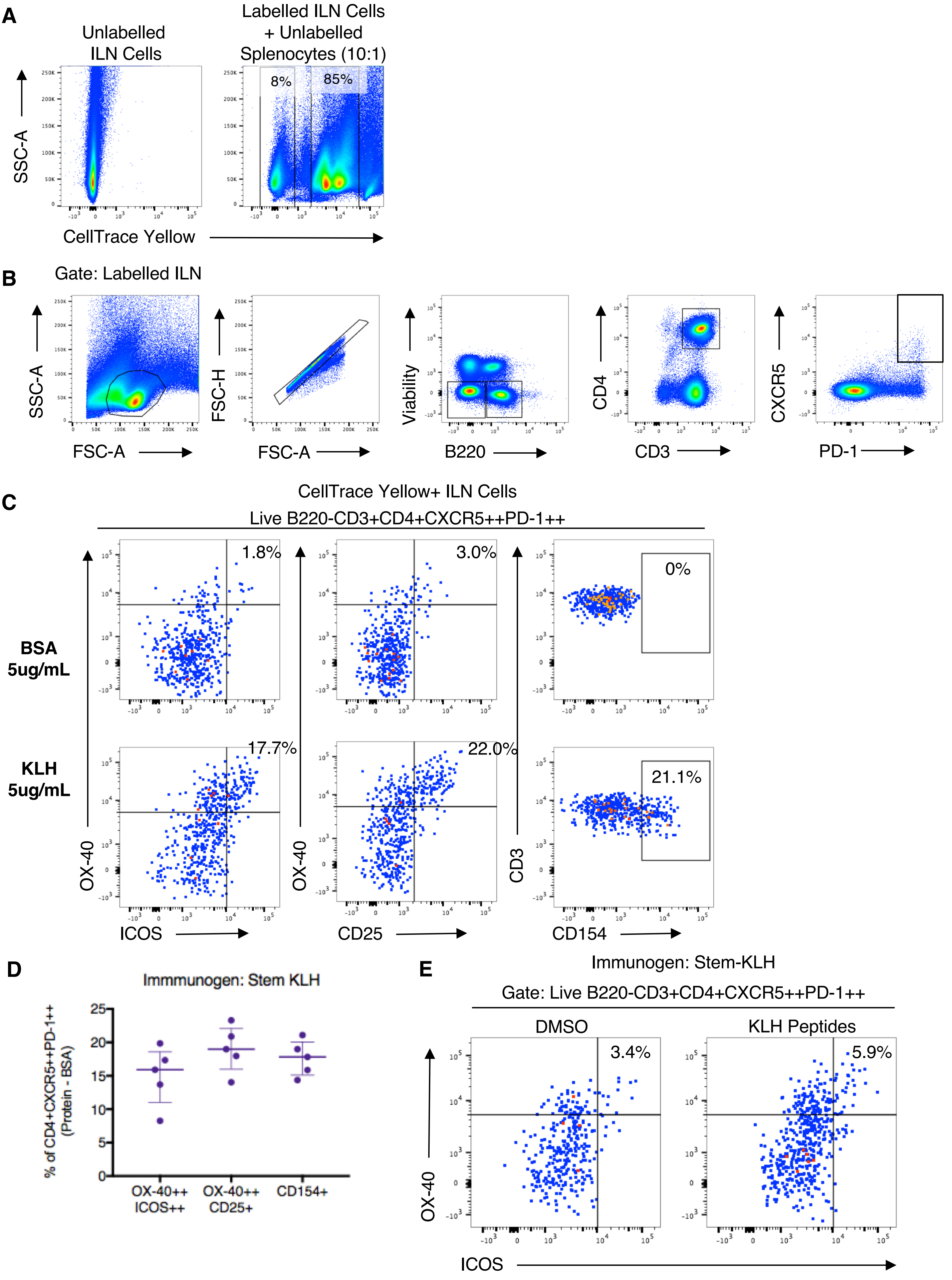
A
Unlabelled ILN Cells
Labelled ILN Cells + Unlabelled Splenocytes (10:1)
8%
85%
SSC-A
CellTrace Yellow
B
Gate: Labelled ILN
SSC-A
FSC-A
FSC-H
FSC-A
Viability
B220
CD4
CD3
CXCR5
PD-1
C
CellTrace Yellow+ ILN Cells
Live B220-CD3+CD4+CXCR5++PD-1++
BSA 5ug/mL
KLH 5ug/mL
1.8%
3.0%
0%
17.7%
22.0%
21.1%
OX-40
ICOS
OX-40
CD25
CD3
CD154
D
Immmunogen: Stem KLH
% of CD4+CXCR5++PD-1++ (Protein - BSA)
OX-40++ ICOS++
OX-40++ CD25+
CD154+
E
Immunogen: Stem-KLH
Gate: Live B220-CD3+CD4+CXCR5++PD-1++
DMSO
KLH Peptides
3.4%
5.9%
OX-40
ICOS

Figure S16. Identification of KLH-specific Tfh cells using a whole protein assay
(A) ILN-derived cells were labelled with CellTrace yellow dye and co-cultured for 18 hours with unlabelled splenocytes at a 10:1 ratio. Plots demonstrate identification of CellTrace yellow+ ILN cells after 18 hours of culture, with unlabelled, cultured ILN cells used as a gating control. **(B)** Tfh cells were identified as previously described, by gating on lymphocytes (FSC-A vs SSC-A), single cells (FSC-A vs FSC-H) and B220- viable cells. Tfh cells were defined as CD3+CD4+CXCR5++PD-1++ cells. **(C)** ILN/splenocyte co-cultures from stem-KLH vaccinated animals were stimulated with 5 μg/mL of either BSA or KLH protein in the presence of anti-CD154 antibody, and the expression of OX-40, ICOS and CD154 was assessed. Plots show a representative KLH-specific response in ILN cells from a single animal. **(D)** KLH-specific Tfh cells were quantified in n = 5 animals at day 14 post-vaccination with stem-KLH protein. **(E)** A pool of 20 15-mer KLH peptides, predicted by Tepitool to be immunogenic in C57BL/6 mice, was used to identify KLH-specific Tfh responses in ILN cells.

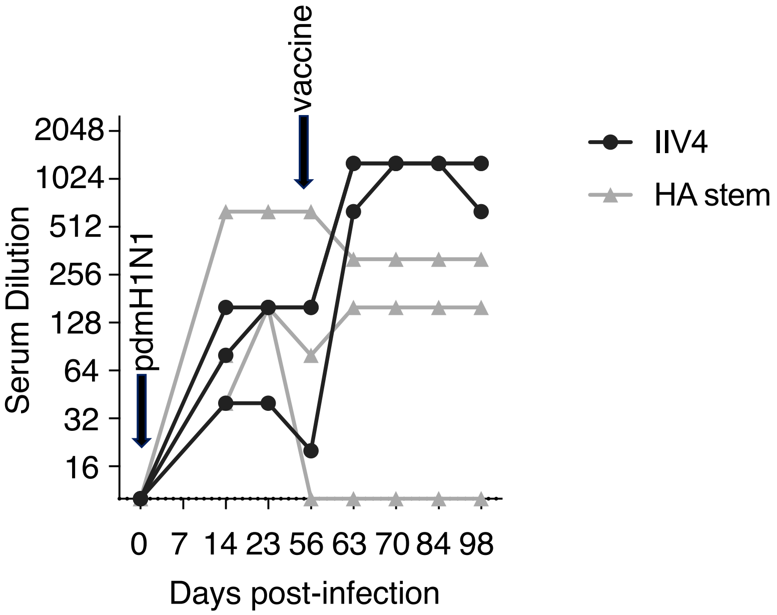

Figure S17. Neutralisation activity against CA09 (focus reduction assay) in the sera of macaques infected with pdmH1N1 (D0) and immunised with IIV4 (n = 3) or HA-stem (n = 3) on day 56.

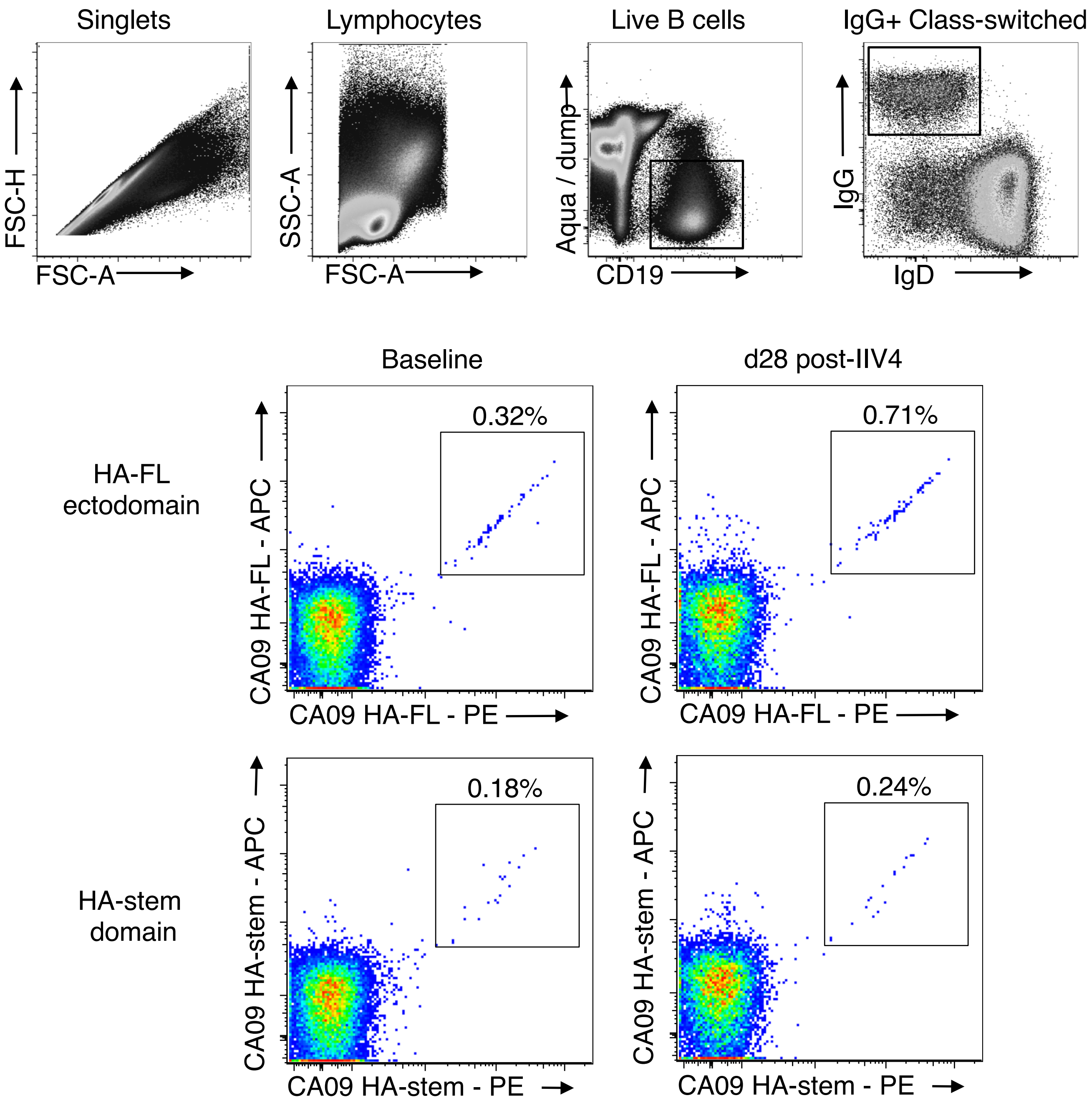

Figure S18. Representative gating scheme of human cryopreserved PBMC stained with HA-FL and HA-stem probes

After doublet exclusion, and dumping of dead cells (Aqua Live Dead) and CD3/8/10/16/14 positive lymphocytes, IgG+ memory B cells (CD19+IgD-IgG+) enumerated based upon binding to recombinant HA probes.

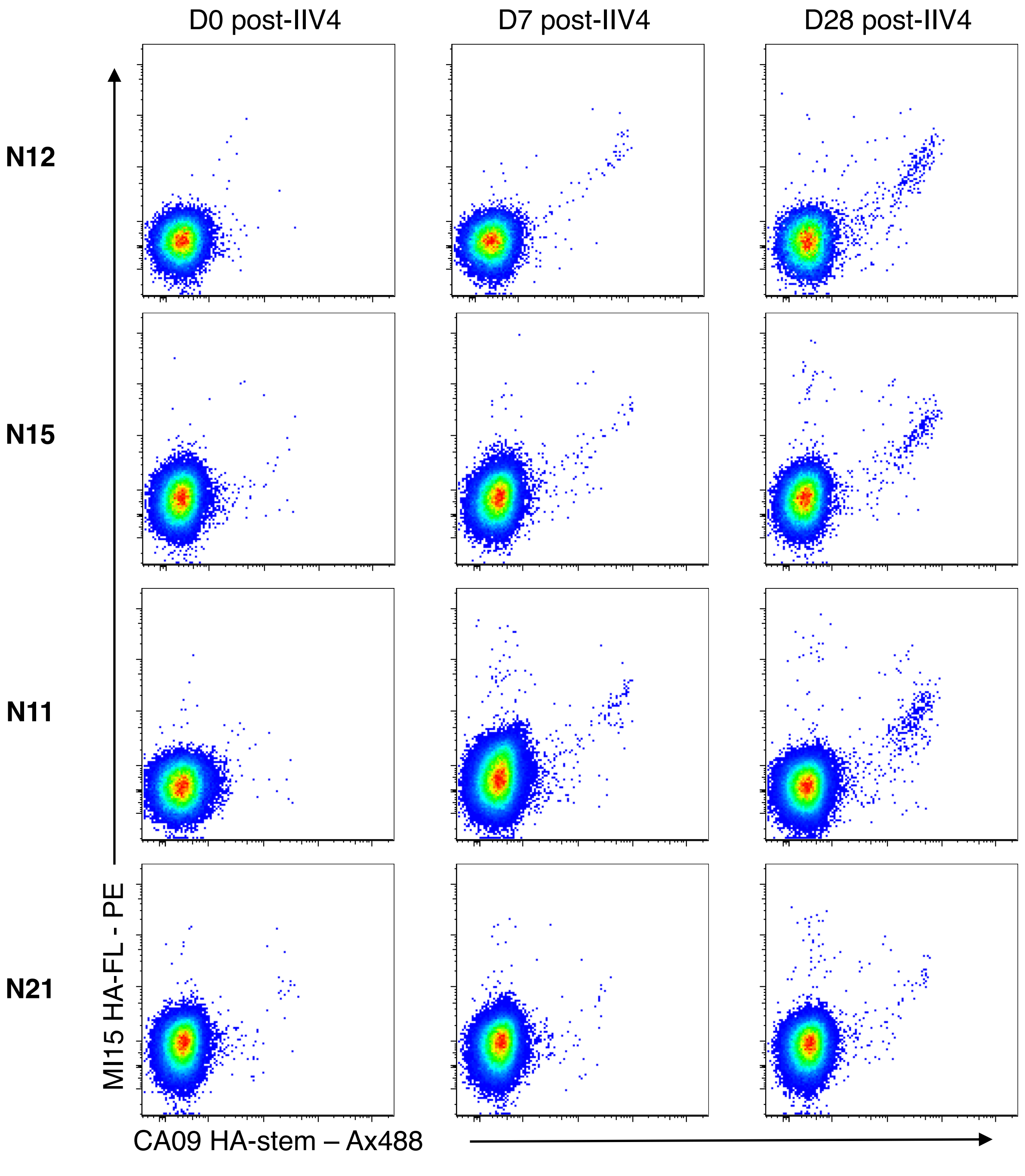

Figure S19. Marked expansion of stem-specific IgG+ memory B cells in selected individuals (4 out of 21 donors shown; N11/12/15/21 denotes donor codes) following immunisation with IIV4 (2017).
